# Ge–Cu-Complexes Ph(pyO)Ge(μ^2^-pyO)_2_CuCl and PhGe(μ^2^-pyO)_4_CuCl—Representatives of Cu(I)→Ge(IV) and Cu(II)→Ge(IV) Dative Bond Systems

**DOI:** 10.3390/molecules28145442

**Published:** 2023-07-16

**Authors:** Jörg Wagler, Robert Gericke

**Affiliations:** 1 Institut für Anorganische Chemie, TU Bergakademie Freiberg, 09596 Freiberg, Germany; 2 Institute of Resource Ecology, Helmholtz-Zentrum Dresden-Rossendorf eV, 01328 Dresden, Germany; gericker.chemie@gmail.com

**Keywords:** atoms-in-molecules, copper, dimetallic complexes, germanium, NLMO, X-ray diffraction

## Abstract

Phenylgermaniumpyridine-2-olate PhGe(pyO)_3_ (compound **1Ge**) and CuCl react with the formation of the heteronuclear complex Ph(pyO)Ge(μ^2^-pyO)_2_CuCl (**2Ge’**) rather than forming the expected compound PhGe(μ^2^-pyO)_3_CuCl (***2Ge***). Single-point calculations (at the B2T-PLYP level) of the optimized molecular structures confirmed the relative stability of isomer **2Ge’** over ***2Ge*** and, for the related silicon congeners, the relative stability of **2Si** over ***2Si’***. Decomposition of a solution of **2Ge’** upon access to air provided access to some crystals of the copper(II) compound PhGe(μ^2^-pyO)_4_CuCl (**3Ge**). Compounds **2Ge’** and **3Ge** were characterized by single-crystal X-ray diffraction analyses, and the Ge–Cu bonds in these compounds were analyzed with the aid of quantum chemical calculations, e.g., Natural Bond Orbital analyses (NBO), Non-Covalent Interactions descriptor (NCI), and topology of the electron density at bond critical point using Quantum Theory of Atoms-In-Molecules (QTAIM) in conjunction with the related silicon compounds PhSi(μ^2^-pyO)_3_CuCl (**2Si**), PhSi(μ^2^-pyO)_4_CuCl (**3Si**), as well as the potential isomers Ph(pyO)Si(μ^2^-pyO)_2_CuCl (***2Si’***) and PhGe(μ^2^-pyO)_3_CuCl (***2Ge***). Pronounced Cu→Ge (over Cu→Si) lone pair donation was found for the Cu(I) compounds, whereas in Cu(II) compounds **3Si** and **3Ge,** this σ-donation is less pronounced and only marginally enhanced in **3Ge** over **3Si**.

## 1. Introduction

**Figure 1:**
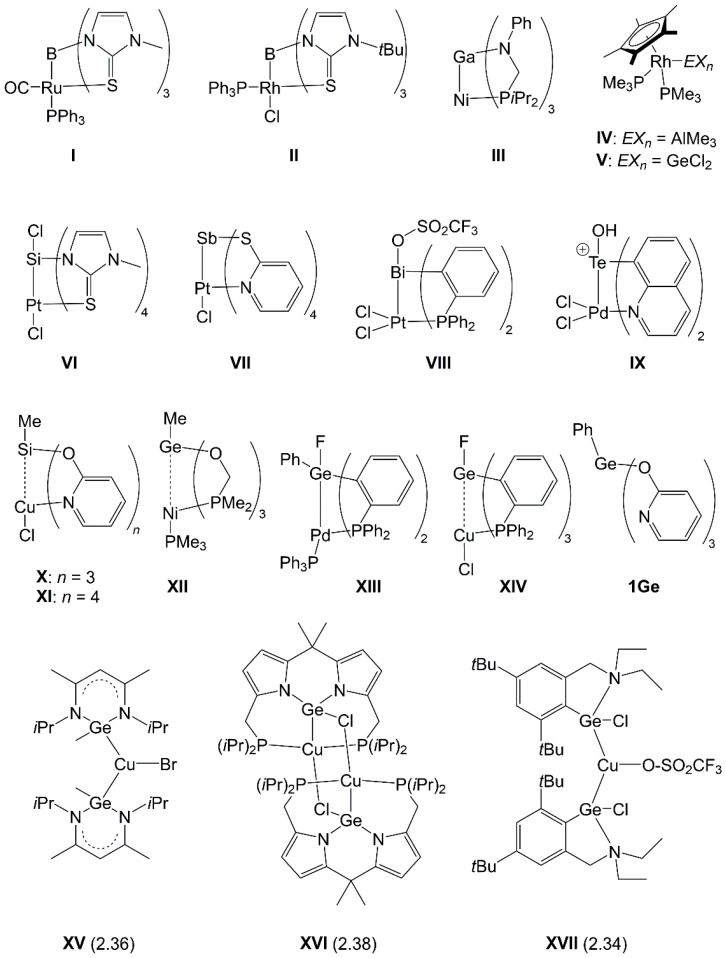
Examples of compounds that feature donor-acceptor bonds between a d-block element donor site and a p-block element acceptor site (compounds **I**–**XIV**) as well as the starting material for the current study (**1Ge**). Note: The donor-acceptor bonds vary in strength and have simply been accounted for by drawing a bond in compounds **I**–**IX**. For the compounds of particular interest in the current study, the presence of rather weak donor-acceptor interactions in **X**–**XII** and **XIV** is underlined by the use of a dashed line rather than a solid bond. Compounds **XV**–**XVII** represent selected examples of Cu–Ge-compounds with germyl- or germylene-type Cu–Ge bonds (Cu–Ge bond length in Å is given in parentheses).

Electron-rich d-block elements may serve as lone pair donors, a feature that is utilized in so-called reverse salt elimination reactions, in which the transition metal complex acts as a metalla-nucleophile, e.g., in the reactions of Na[Co(CO)_4_] or Na[FeCp(CO)_2_] and a chlorosilane [1]. In a similar manner, they may serve as anionic ligands, e.g., upon adding Na[FeCp(CO)_2_] to a spirocyclic Ge(IV) compound [2]. In general, in coordination chemistry, d-block metal located nucleophilic lone pairs can bind Lewis acidic sites (so-called Z-type ligands [3]) in the transition metal coordination sphere. So-called metallaboratranes (such as **I** [4] and **II** [5], Figure 1), related complexes with other group 13 Z-type ligand sites (such as **III** [6] and **IV** [7]), and d-block element complexes with Z-type ligand sites from groups 14 (e.g., **V** [7] and **VI** [8]), 15 (e.g., **VII** [9] and **VIII** [10]), and 16 (e.g., **IX** [11]) were reported. In principle, d^10^ metal sites are suited as lone pair donors, which is reflected by compounds such as (Cy_3_P)_2_PtBeCl_2_ [12], in which a Pt→Be bond is contained and relatively stable even without the aid of buttressing ligands (which is a common feature of most of the compounds **I**–**IX**).

In the context of exploring group 14 compounds (especially Si compounds) as lone pair acceptor ligands in electron-rich d-block element complexes, we succeeded in generating Cu(I) and Cu(II) complexes, which feature short Cu⋯Si contacts [13,14], and Natural Localized Molecular Orbital (NLMO) analyses of compounds **X** and **XI** [13] indicated weak Cu→Si lone pair donation. Silicon’s heavier congener, germanium, is only scarcely explored as a Z-type ligand site (e.g., Ge(II) compound **V** or Ge(IV) compounds **XII** [15] and **XIII** [16]). A search in the Cambridge Structure Database (accessed on 9 June 2023, see Appendix A) [17] yielded a single hit for a heterodinuclear (Cu, Ge) compound, which may accommodate a Cu→Ge dative bond. This compound (**XIV**) features a Cu−Ge distance of 3.372(1) Å, and the authors identified weak Cu→Ge σ-donation (1.77 kcal mol^−1^) in a second-order perturbation of a Natural Bond Orbital (NBO) analysis [18]. For comparison, this Cu−Ge distance is about 1 Å longer than “regular” Cu−Ge single bonds in Cu(I)-germyl- or Cu(I)-germylene complexes (e.g., **XV** [19], **XVI** [20], and **XVII** [21]). With the starting material PhGe(pyO)_3_ (pyO = pyridine-2-olate), compound **1Ge**, available [22], we addressed the exploration of compounds with Cu(I)→Ge and Cu(II)→Ge bonding in the following study.

## 2. Results and Discussion

### 2.1. Syntheses (Compounds Overview)

**Scheme 1:**
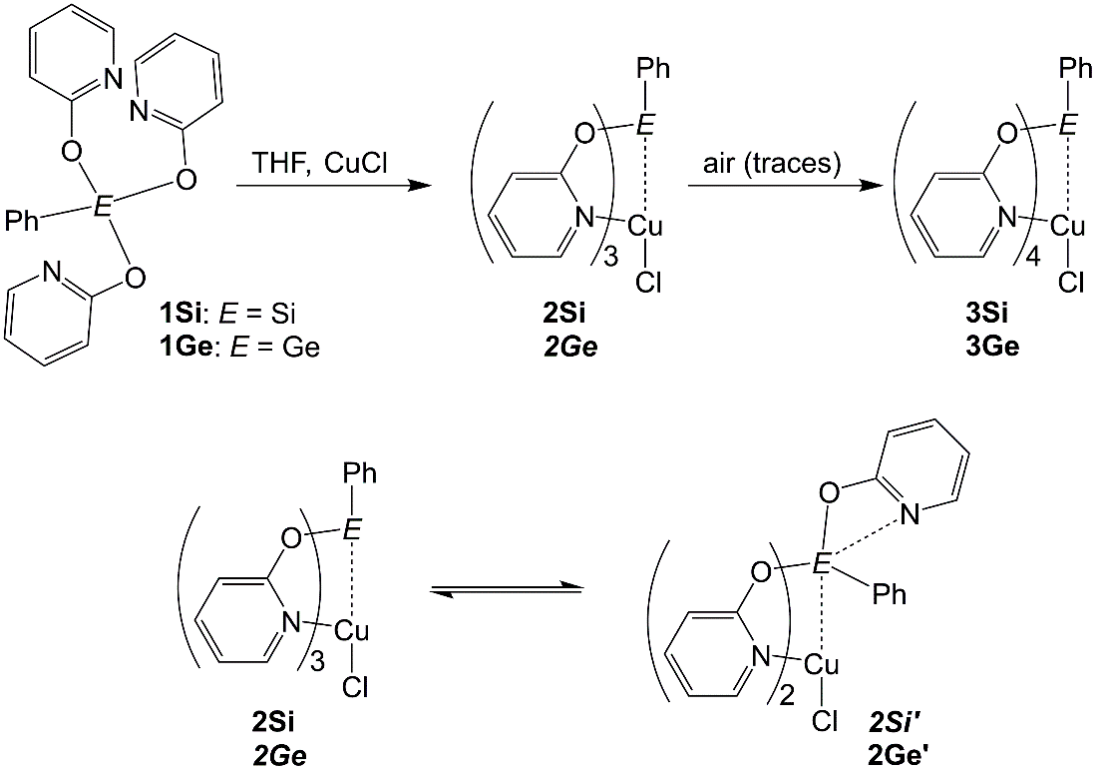
Syntheses of the compounds under investigation. (The compound codes of the potential isomers ***2Ge*** and ***2Si’***, which have not been isolated or identified experimentally but will be included in computational analyses, are italicized.)

The silicon compounds **1Si** [22], **2Si,** and **3Si** [14] were published earlier; Cu–Si compounds **2Si** and **3Si** will serve as reference compounds for computational analyses (cf. Section 2.3). Germane **1Ge** was available from a previous study [22] and was treated with CuCl in chloroform (Scheme 1). Upon layering the resultant solution with diethyl ether, colorless crystals formed, which were suitable for single-crystal X-ray diffraction analysis (cf. Section 2.2). Rather than the expected Ge-analog of **2Si**, a compound ***2Ge***, the analysis revealed the formation of an isomer **2Ge’** (Scheme 1), which features two bridging pyO ligands and one terminal pyO group at the Ge atom. The ^1^H and ^13^C NMR spectra of this compound (cf. Figures S1 and S2) resemble those of the silicon compound **2Si** [14] (cf. Figures S3 and S4) and are indicative of conformational mobility in solution. Access of traces of air to the crude solution of Cu(I) complexes (presumably containing **2Ge’** and possible isomers thereof) resulted in a color change of the solution (from near colorless to deep blue) and the formation of some dark blue crystals, which were also suitable for single-crystal X-ray diffraction. They revealed the formation of the Ge-analog (**3Ge**) to the silicon compound **3Si** (cf. Section 2.2). To the best of our knowledge, compounds **2Ge’** and **3Ge** represent the first crystallographically characterized Cu–Ge compounds that feature a Cu→Ge dative bond shorter than 3 Å [17]. Quantum chemical calculations will address both the analyses of the relative stabilities of the isomers **2*E*** vs. **2*E*’** (for *E* = Si, Ge) and the comparison of the Cu→*E* dative bonds in compounds **2Si**, ***2Ge***, ***2Si’***, **2Ge’**, **3Si,** and **3Ge** (cf. Section 2.3).

### 2.2. Single-Crystal X-ray Diffraction

**Figure 2:**
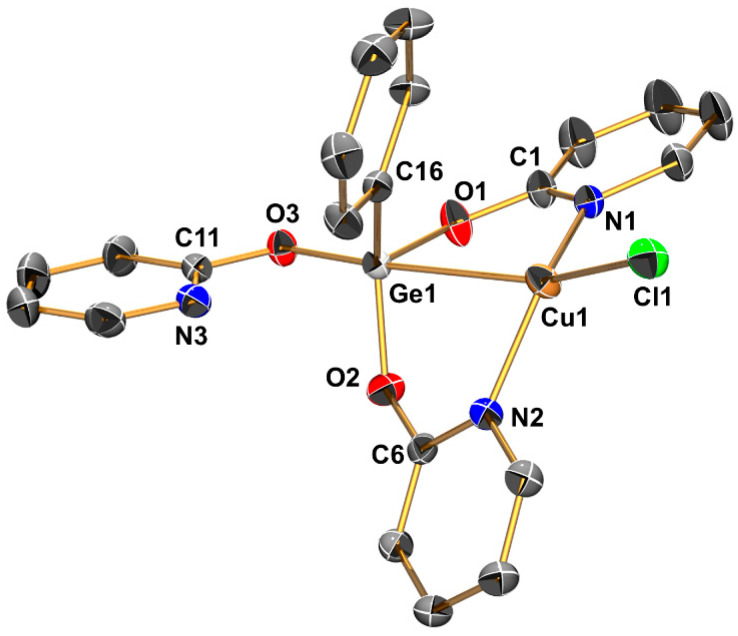
Molecular structure of **2Ge’** in the crystal (displacement ellipsoids drawn at the 50% probability level, selected atoms labeled; H atoms are omitted for clarity). Selected interatomic distances (Å) and angles (deg.): Ge1–Cu1 2.7886(3), Ge1–O1 1.7919(14), Ge1–O2 1.7794(14), Ge1–O3 1.7894(14), Ge1⋯N3 2.6840(17), Ge1–C16 1.9217(19), Cu1–Cl1 2.2669(5), Cu1–N1 1.9767(16), Cu1–N2 1.9782(16), Cu1⋯C16 2.6044(18), Cu1–Ge1–O1 84.23(5), Cu1–Ge1–O2 83.63(4), Cu1–Ge1–O3 174.78(5), Cu1–Ge1–C16 64.07(5), O1–Ge1–O2 104.58(7), O1–Ge1–O3 90.72(6), O2–Ge1–O3 98.94(6), O1–Ge1–C16 112.66(8), O2–Ge1–C16 126.41(7), O3–Ge1–C16 117.10(7), O1–Ge1–N3 147.52(6), Ge1–Gu1–Cl1 156.38(2), Cl1–Cu1–N1 108.80(5), Cl1–Cu1–N2 106.63(5), N1–Cu1–N2 135.69(7).

Compound **2Ge’** crystallized in the monoclinic space group *P*2_1_/*n* with one molecule in the asymmetric unit (Figure 2, Table A1). The Ge–Cu core is bridged by two of the three pyO ligands, and the distance Ge1–Cu1 (2.7886(3) Å) is markedly shorter than the Si–Cu-distance in the three-fold pyO-bridged compound **2Si** (3.211(1) Å [14]). This indicates a pronounced Ge–Cu attractive interaction and will be analyzed further in Section 2.3. As a result of the Cu atom in the Ge coordination sphere (with reference to an inherently tetrahedral Ge(C,O,O,O) coordination), the sum of angles spanned by C16, O1, and O2 amounts to 343.7°, which is midway between the three angles of bonds in a tetrahedral coordination sphere (328.5°) and within a planar arrangement (360°). In this context, the rather sharp angle Cu1–Ge1–C16 (64.07(5)°) is noteworthy; it hints at attractive interactions between the Cu atom and the Ge-bound phenyl group. (This arrangement resembles the rather sharp Pd–Ge–C_ipso_ angle of 65.7° in compound **XIII** [16], which features Pd(0) as a d^10^-ML_3_ donor site in the Ge coordination sphere.) The resultant Cu⋯C_ipso_ distance (2.6044(18) Å) is surprisingly short. Also, the Ge atom is slightly displaced out of the phenyl group’s least-squares plane (by 0.074(3) Å), which indicates an attraction of the phenyl group with its C_ipso_ toward the Cu atom. A search in the Cambridge Structure Database (CSD, accessed on 9 June 2023) [17] did not deliver any hits for aryl-Ge compounds with Cu⋯C_ipso_ contacts shorter than 3 Å, and even for phenyl-silicon-compounds, the two shortest Cu⋯C_ipso_ contacts available in the database are ca. 2.95 Å, i.e., for a dimeric *N*-heterocyclic carbene (NHC) supported Cu(I) silanide [(NHC)Cu(μ^2^-SiPh_3_)]_2_ [23] and for a tetrameric Cu(I) silanethiolate [Cu(μ^2^-SSiPh_3_)]_4_ [24].

Furthermore, the tetrahedral face spanned by C16, O2, and O3 is also widened to a similar extent (sum of angles 342.5°). This is caused by atom N3, which is capping this face at an N⋯Ge distance of 2.684(2) Å. This remote N⋯Ge coordination is more pronounced than in the starting material **1Ge**, which features a [4+3] Ge coordination sphere with N⋯Ge distances of 2.789(2), 2.908(2), and 2.923(2) Å [22]. As the two capped tetrahedral faces share atoms O2 and C16, angle O2–Ge1–C16 (126.41(7)°) is the widest angle in the Ge(C,O,O,O) coordination sphere of **2Ge’**, and the opposite angle (O1–Ge1–O3 90.72(6)°) is the smallest. Furthermore, the remote coordination of Ge1 by Cu1 and N3 causes some lengthening of the *trans*-bonds Ge1–O3 and Ge1–O1, respectively, which are ca. 0.01 Å longer than the bond Ge1–O2.

The Cu coordination sphere of **2Ge’** is tetracoordinated in a highly distorted tetrahedral manner (bisphenoidal coordination). This is also expressed by the geometry index *τ*_4_ [25], which is 0.48 for this Cu coordination sphere. The *trans*-angles (with respect to an idealized square-planar coordination, N1–Cu1–N2 135.69(7)°, Ge1–Cu1–Cl1 156.38(2)°) are markedly bent out of linearity but wider than the tetrahedral angle. This Cu coordination sphere is accompanied by shorter Cu–N bonds (ca. 1.98 Å in **2Ge’** vs. ca. 2.04 Å in **2Si**) and a shorter Cu–Cl bond (ca. 2.2669(5) Å in **2Ge’** vs. ca. 2.359(1) Å in **2Si**) with respect to the three-fold pyO-bridged CuCl complex **2Si** [14], which has a rather tetrahedral Cu(Cl,N,N,N) coordination sphere. In **2Ge’**, the Cu(Cl,N,N) coordination sphere is markedly planarized, which is indicated by the sum of angles of 351.1°, and distorted out of trigonality, which is indicated by the N–Cu–N angle (135.69(7)°) being much wider than the Cl–Cu–N angles (108.80(5) and 106.63(5)°). This moiety itself reflects the transition of a d^10^-ML_3_ system from a trigonal-planar toward a T-shape coordination, which also indicates the development of a nucleophilic lone pair, a prerequisite for Cu→Ge coordination. This T-shape coordination of d^10^-ML_3_ sites, which bind to a Lewis-acidic fourth site, is known from other heterodinuclear complexes, e.g., with Hg(II)→Sb(V) [26], Au(I)→Sb(V) [27], Pd(0)→Si(IV) [28], Pd(0)→Ge(IV) [16], and Au(I)→Sn(IV) cores [29].

**Figure 3:**
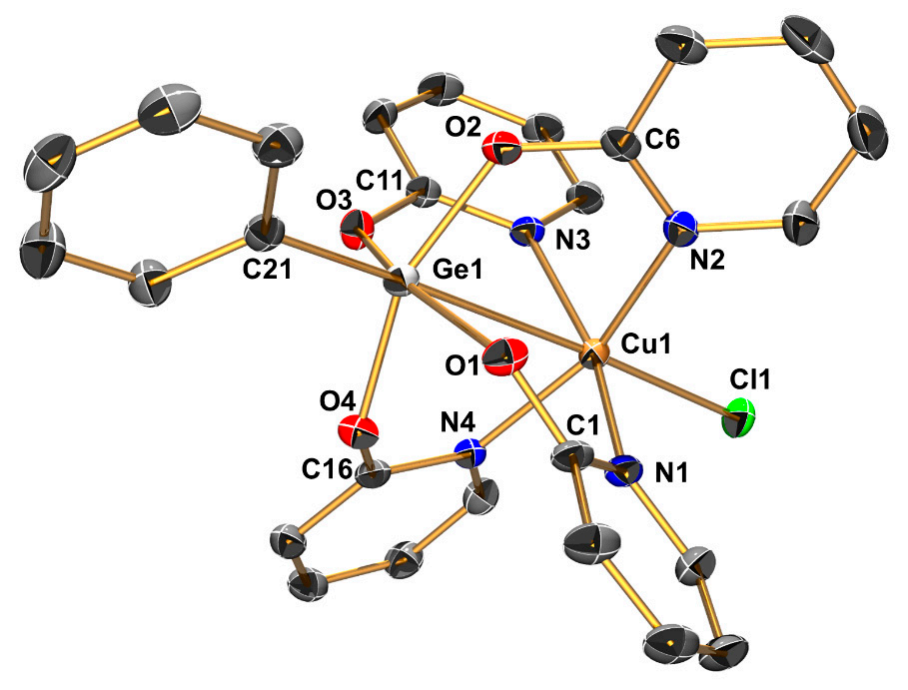
Molecular structure of **3Ge** in the crystal (displacement ellipsoids drawn at the 50% probability level, selected atoms labeled; H atoms are omitted for clarity). Selected interatomic distances (Å) and angles (deg.): Ge1–Cu1 2.8969(4), Ge1–O1 1.8856(18), Ge1–O2 1.8543(19), Ge1–O3 1.8785(18), Ge1–O4 1.8691(18), Ge1–C21 1.915(3), Cu1–Cl1 2.4027(7), Cu1–N1 2.043(2), Cu1–N2 2.033(2), Cu1–N3 2.027(2), Cu1–N4 2.034(2), Cu1–Ge1–C21 177.85(8), O1–Ge1–O3 155.96(8), O2–Ge1–O4 154.10(8), Ge1–Cu1–Cl1 177.59(2), N1–Cu1–N3 161.77(9), N2–Cu1–N4 161.61(9).

Compound **3Ge** crystallized in the orthorhombic space group *Pbca* with one molecule in the asymmetric unit (Figure 3, Table A1). The crystal structures of **3Ge** and of the related silicon compound **3Si** [14] are isomorphous; they crystallize in the same space group type, and their unit cell axes are nearly identical (**3Ge**: 15.730, 18.255, 17.179 Å, **3Si** 15.756, 18.257, 17.201 Å for axes *a*, *b*, and *c*, respectively). (Moreover, the crystal structure of **3Ge** is isomorphous with those of compounds PhSb(μ^2^-pyO)_4_Ru(CO) and PhSb(μ^2^-pyO)_4_RuCl [30], as has been noticed for **3Si** before [14]). The molecular conformation of **3Ge** essentially resembles that of **3Si**, and their Cu–N and Cu–Cl bond lengths are very similar. The largest differences are associated with the Ge vs. Si coordination spheres because of the different covalent radii of these group 14 elements (Ge: 1.20(4), Si: 1.11(2) Å [31]). Whereas in **3Si** the Si–O bond lengths are ca. 1.75 Å, the Ge–O bond lengths in **3Ge** are ca. 1.87 Å, and the Ge–C bond is ca. 0.05 Å longer than the corresponding Si–C bond. The longer Ge–O bonds (and the expansion of the molecule associated with the pyO oxygen atoms) give rise to slightly wider N–Cu–N angles (ca. 159.6° in **3Si**, 161.7° in **3Ge**). Noteworthy, in spite of the larger covalent radius of Ge (relative to Si), the Cu–*E* (*E* = Si, Ge) distances in the dinuclear cores of **3Si** (2.888(1) Å) and **3Ge** (2.8969(4) Å) are very similar. Computational analyses have been employed (cf. Section 2.3.) to answer the question as to whether the absence of interatomic interactions (neither attractive nor repulsive) at this distance is the origin of this similarity or if enhanced attractive interactions in **3Ge** are present and may serve as compensation for the enhanced steric repulsion by the larger Ge atom.

### 2.3. Computational Analyses

#### 2.3.1. Relative Stability of Configurational Isomers **2Si** vs. ***2Si’*** and ***2Ge*** vs. **2Ge’**

**Figure 4:**
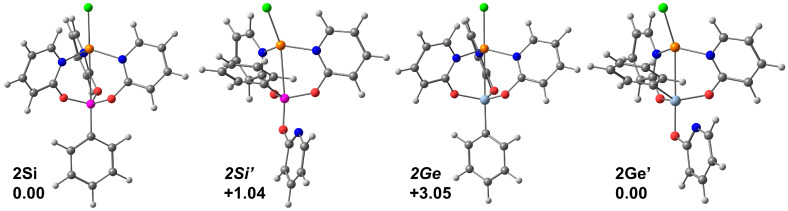
Pairs of isomers **2Si**/***2Si’*** and ***2Ge***/**2Ge’** optimized at the PBE0 level of theory and their relative energy values in kcal mol^−1^ from single-point calculation at the B2T-PLYP level.

The question regarding the relative stabilities of **2Si** and its potential isomer ***2Si’*** as well as of the pair **2Ge’** and its potential isomer ***2Ge*** was addressed with optimized molecular structures of these compounds, which used the solid-state molecular structures of **2Si** and **2Ge’** as a starting point for generating the isomers ***2Ge*** and ***2Si’***, respectively, by replacing the central group 14 element type. Optimization of their molecular conformations in a solvent environment model (COSMO) for chloroform afforded the molecules shown in Figure 4, which were confirmed as local minima on the potential energy hypersurface by frequency analyses. In principle, the optimized molecular structures of **2Si** and **2Ge’** resemble the experimentally found molecular conformations very well, and the conformations within the pairs of congeners, i.e., **2Si**/***2Ge*** and ***2Si’***/**2Ge’**, are similar to one another. For compound **2Ge’**, the interesting feature of the close Cu⋯C_ipso_ contact (2.601 Å) is still present in the optimized molecular structure, and the related silicon compound ***2Si’*** exhibits this feature as well (with a Cu⋯C_ipso_ distance of 2.511 Å). Single-point energy analyses at the B2T-PLYP level indicated for the silicon compound that isomer **2Si** is slightly more stable than isomer ***2Si’*** (by ca. 1 kcal mol^−1^) in accordance with the experimental result [14]. For the germanium compound, the crystallographically found isomer **2Ge’** was confirmed to be more stable (by ca. 3 kcal mol^−1^) than the alternative isomer ***2Ge***.

#### 2.3.2. Non-Covalent Interactions Descriptor (NCI) and Electron Localization Function (ELF)

**Figure 5:**
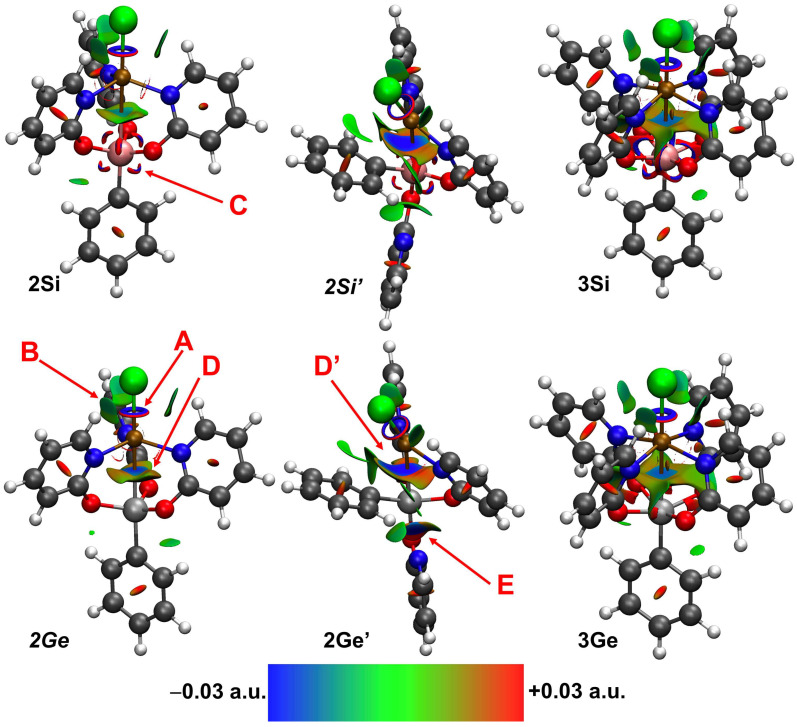
Non-Covalent Interactions descriptor (NCI) for pairs of congeners **2Si**/***2Ge***, ***2Si’***/**2Ge,’** and **3Si**/**3Ge** with color scale (RDG iso-value 0.45; blue zones indicate attractive interactions, red zones indicate repulsive interactions). Particular features (**A**–**E**), which are mentioned in the discussion, are pointed out with red arrows.

For the optimized molecular structures of **2Si**, ***2Si’***, ***2Ge***, and **2Ge’** (cf. Section 2.3.1), as well as for **3Si** and **3Ge** (which were optimized at the same level of theory), the Non-Covalent Interactions descriptor (NCI) was calculated (Figure 5). The six molecules exhibit some common features of their NCI with respect to the environment of the Cl atom. In each case, the Cu–Cl bond is surrounded by a toroid zone, which could be an artifact rather than a genuine NCI feature (feature **A**). Furthermore, patches of attractive interactions are found between the Cl atoms and their adjacent H atoms of the pyO bridging ligands (C–H⋯Cl contacts, feature **B**). A noticeable difference between the set of Si compounds and their Ge congeners is reflected by feature **C**, which can also be interpreted as an artifact rather than a genuine NCI feature (similar to feature **A**), and it will not be discussed further. With respect to the interaction of Cu and the heavier tetrel (Si or Ge), feature **D** is of interest. For all six compounds under investigation, it indicates the presence of non-covalent attractive interactions. Whereas for the Cu(II) compounds **3Si** and **3Ge,** this feature is similar, the pair of Cu(I) compounds **2Si**/***2Ge*** exhibit a pronounced attraction for this feature in the germanium compound. This non-covalent attraction along the Cu-tetrel axis is particularly pronounced for the pair ***2Si’***/**2Ge’**, with the peculiarity that this patch of the attractive zone extends into the interspace of the Cu atom and the tetrel-bound phenyl group (feature **D’**). The latter two compounds exhibit another feature (**E**), which reflects attractive forces between the tetrel and the N atom of the terminal pyO group, which is capping one face of the tetrel’s coordination sphere. Once again, this attractive feature is more pronounced for the germanium congener. Combinations of features **D’** (stronger Cu-tetrel attraction in isomers ***2Si’*** and **2Ge’**) and **E** (stronger coordination of a pyO nitrogen atom to the Ge atom) provide a first insight into the origin of the enhanced stability of isomer **2Ge’** over ***2Ge***. Whereas features **A** and **C** are less interesting for our discussion, features **B**, **D**, **D’**, and **E** are typical weak interactions identified from the so-called Reduced Density Gradient (RDG) function.

**Figure 6:**
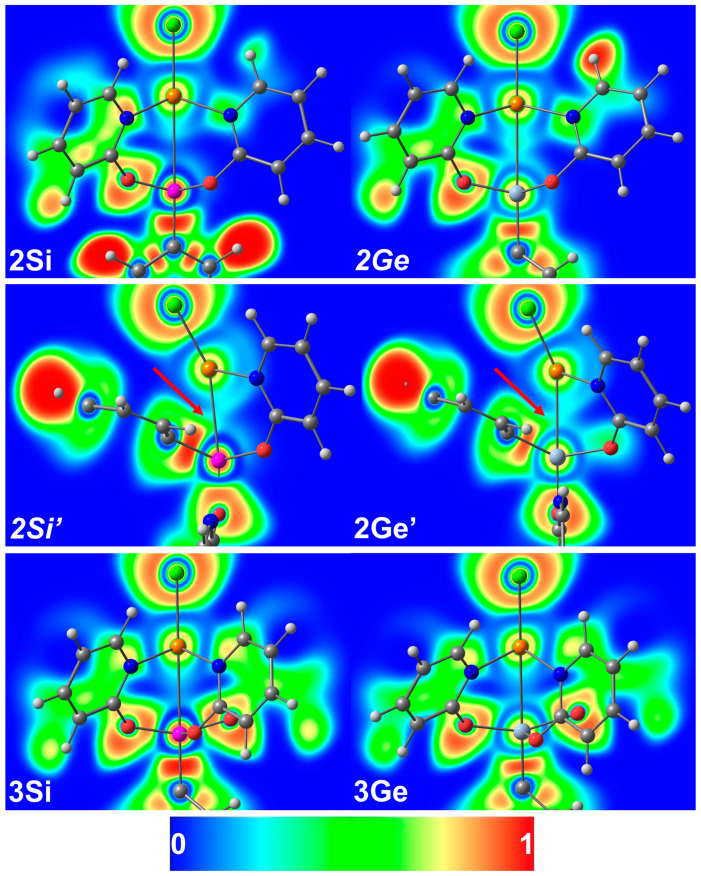
Graphical representation of the Electron Localization Function (ELF) for pairs of congeners **2Si**/***2Ge***, ***2Si’***/**2Ge’,** and **3Si**/**3Ge** with color scale. In all graphics, the Cu–*E* (*E* = Si, Ge) bond is in the plane. For compounds ***2Si’*** and **2Ge’**, the phenyl ipso-C is located in the plane as well. Red arrows point at this atom’s influence on the electron localization near the Cu–*E* (*E* = Si, Ge) bond.

The function *sign*(*λ*_2_(**r_b_**))*ρ*(**r_b_**) allows for a more detailed interpretation of these non-covalent attractive interactions. Whereas values of *sign*(*λ*_2_(**r_b_**))*ρ*(**r_b_**) close to 0 (−0.01…+0.01) are likely speaking for van der Waals interactions, markedly more negative values indicate attractive interactions such as H bonds. Thus, feature **B** (green color, biased toward light blue) is speaking for attractive C–H⋯Cl van der Waals interactions (H contacts). Cu–Si interaction in compound **2Si** is just slightly more attractive (underlined by the value of *sign*(*λ*_2_(**r_b_**))*ρ*(**r_b_**) − 0.014, Table 1). It becomes more attractive in nature for compounds such as **3Si**, **3Ge,** and ***2Ge***. For the former two, for which the graphical NCI representation looks similar, *sign*(*λ*_2_(**r_b_**))*ρ*(**r_b_**) indicates a slightly pronounced attraction for **3Ge** over **3Si**. The values listed for compounds **2*E*’** clearly reveal the pronounced Cu⋯*E* attraction in this class of compounds. As pointed out in Table 1, the reference point (bond critical point (BCP) of the bond path) deviates from the idealized Cu–*E* bond axis in these two compounds. The Electron Localization Function (ELF) shows that the tetrel-bound phenyl group contributes to the electron localization near the Cu–*E* bond (Figure 6) and causes a shift of this reference point off the intuitive bond axis. This effect also influences the location of the predominant non-covalent attraction within the dinuclear cores of these compounds.

The other trends observed with feature **D** of the NCI (cf. Figure 5) are also reflected by the ELF in Figure 6. The stronger Cu⋯Ge attraction in ***2Ge*** (over Cu⋯Si attraction in **2Si**) is accompanied by enhanced electron localization on the Cu–Ge bond axis. For the pair of compounds **3Si** and **3Ge**, the ELF on the Cu–*E* bond axes are similar to one another and indicate electron localization, which is less pronounced than in compound ***2Ge*** (Table 2, Section 2.3.3).

#### 2.3.3. Topological Analysis with Quantum Theory of Atoms-in-Molecules

To supplement the insights from Section 2.3.2, a Quantum Theory of Atoms-In-Molecules (QTAIM) analysis was performed for the compounds **2Si**, ***2Si’***, ***2Ge***, **2Ge’**, **3Si,** and **3Ge**. Table 2 lists selected characteristic features for selected bonds at their (3, −1) critical points (i.e., bond critical points, BCPs). For compounds **2Si**, ***2Ge***, **3Si,** and **3Ge**, the BCPs of interest were detected between the Cu and the Si or Ge atom on the intuitive bond axis. For compounds ***2Si’*** and **2Ge’**, corresponding BCPs were detected off the Cu–*E* (*E* = Si, Ge) axis (cf. Figure S11 in the Supporting Information). Whereas the ELF on the Cu–*E* bond axes, in general, is most pronounced for compound ***2Ge*** (see Figure 6), this is also observed at the corresponding BCP of this compound, and the BCP (off the axis) of compound **2Ge’** exhibits similar ELF features. The electron density itself at the BCP is highest for compounds **2Ge’** and, in particular, ***2Si’***. This discrepancy can be explained by the offset of the BCP in the latter two compounds toward the phenyl group, thus gaining electron density from the proximity to the electron system of this moiety. The pronounced ellipticity of the electron density at the BCP for compounds **2Ge’** and ***2Si’*** underlines the influence of this additional source of electron density, which disturbs the more radially symmetric features expected for a Cu–*E* σ-bond. In accordance with these pronounced features of the electron density and ELF, the Cu–*E* interactions in compounds ***2Ge***, ***2Si’,*** and **2Ge’** exhibit the highest Wiberg Bond Index (WBI) and the highest total Delocalization Index (DI). (The latter can range between 0 for perfect ionic interactions without shared electron density and 1 for perfectly covalent single bonds with equal contributions of the atoms involved). In general, the WBI in the range of 0.1–0.3 speaks for rather weak bonding interactions, and the DI indicates rather one-sided lone-pair donation. In accordance with these features (low electron density at the BCP, DI close to zero, low WBI) and the non-covalent interactions identified in Section 2.3.2., the Laplacian along the bond path (cf. Supporting Information Figure S13) is indicative of closed-shell interactions rather than of dative bonds with pronounced covalent character [32]. In this regard, the ratio of the modulus of the potential energy density per Lagrangian kinetic energy density is in the range 1 < |*V*(**r_b_**)|/*G*(**r_b_**) < 2 in all cases and is indicative of an intermediate bond characteristic (i.e., a closed-shell covalent bond with additional ionic contribution).

#### 2.3.4. NBO-/NLMO-Analyses

For a closer look at the individual atomic contributions to the Cu–*E* bonds in compounds **2Si**, ***2Si’***, ***2Ge***, **2Ge’**, **3Si,** and **3Ge**, analyses of Natural Bond Orbitals (NBOs) and Natural Localized Molecular Orbitals (NLMOs) were performed. As the set of compounds under investigation includes open-shell systems (the Cu(II) compounds **3Si** and **3Ge**), all calculations were performed for open systems for the sake of consistent treatment. As expected, for the closed shell compounds **2Si**, ***2Si’***, ***2Ge,*** and **2Ge’,** the α- and β-spin contributions to NBO and NLMO populations were identical, whereas, for the Cu(II) compounds **3Si** and **3Ge,** individual α- and β-spin contributions of different magnitude to NBO and NLMO populations were obtained. Thus, in Table 3, the individual α- and β-spin contributions are listed for compounds **3Si** and **3Ge.** In principle, the α- and β-spin contributions are rather similar, and for comparison with the data of compounds **2Si**, ***2Si’***, ***2Ge,*** and **2Ge’,** the average values are listed as well.

The Natural Charges (NCs) of the heavy tetrel atoms in these compounds vary in a narrow range only (2.11–2.25), with the trend of the Si atoms carrying a slightly more positive charge than the Ge atoms. The different oxidation number of Cu, however, is reflected by the more positive NC of Cu in Cu(II) compounds **3Si** and **3Ge**. In contrast, the different coordination numbers or coordination spheres’ geometries do not hint at any trends.

In all cases, the Cu–*E* bonds can be described as localized two-electron-two-atom interactions. The slightly lower sum of diatomic contributions in the case of compounds ***2Si’*** (97.2%) and **2Ge’** (97.6%) indicates the influence of further atoms. As a mutual feature, the Cu atom contributes the σ-bond electron pair, whereas the tetrel is the Lewis acceptor. For Cu(I) compounds **2*E*** and **2*E*’**, the tetrel is more involved in bond formation when *E* = Ge. For the Cu(II) compounds, this trend is still reflected, but to a marginal extent.

**Figure 7:**
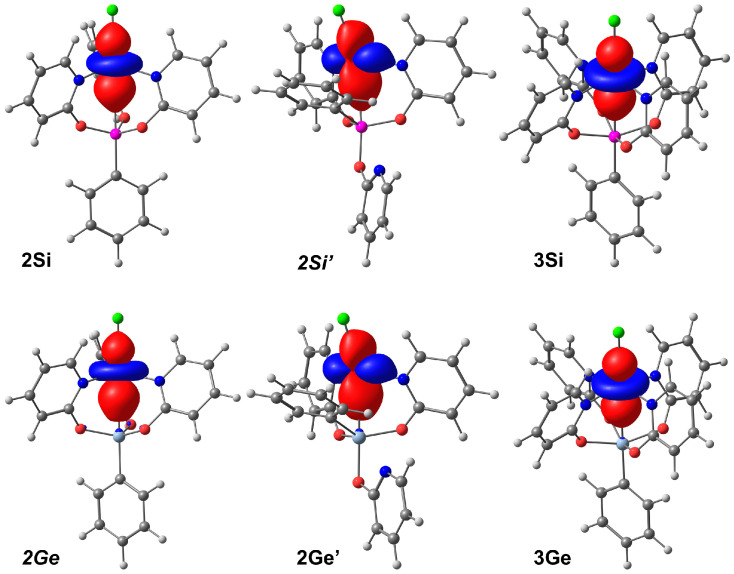
Visualization of NLMOs (isosurface 0.05 au) involved in σ-Cu–*E* bonding in compounds **2Si**, ***2Si’***, ***2Ge***, **2Ge’**, **3Si,** and **3Ge**.

Figure 7 shows the β-spin contributions of the six Cu–*E* σ-bond NLMOs. (For compounds **3Si** and **3Ge**, the α-spin contributions are similar, they are shown in the Supporting Information, Figure S12). Whereas for compounds **2*E*** and **3*E,*** these NLMOs reflect the interaction of the Cu 3d(z^2^) orbital as a lone-pair donor with the Lewis-acidic tetrel site, in compounds **2*E*’**, the donor orbital resembles the Cu 3d(x^2^ − y^2^) orbital, which is in accordance with the expected Cu-located nucleophilic lone-pair for a rather T-shaped d^10^-ML_3_ coordination sphere.

**Figure 8:**
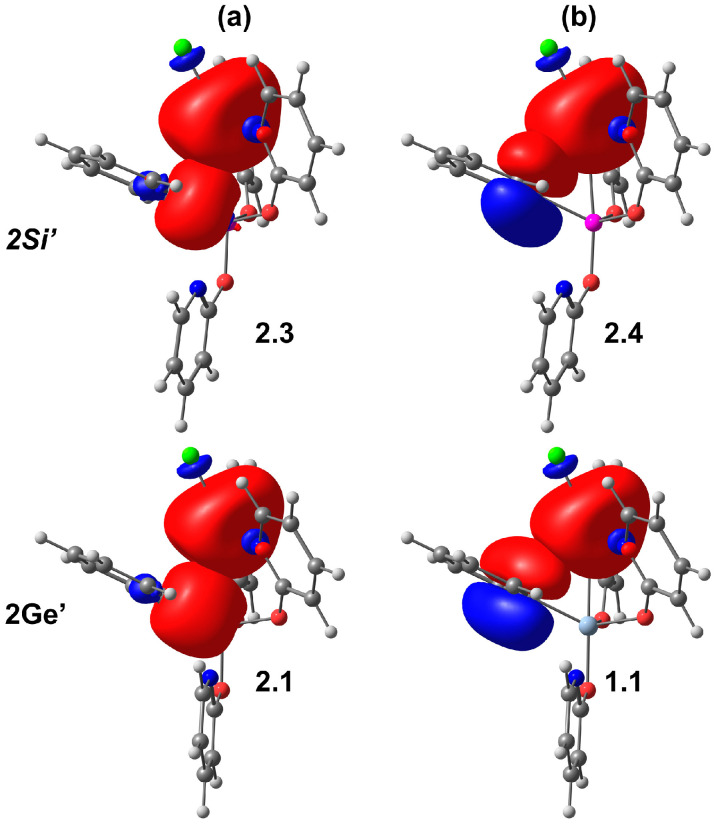
Visualization of NBOs (isosurface 0.05 au) involved in electron density donation from the tetrel-bound phenyl group (from the tetrel−C_ipso_ bond (**a**) and from a phenyl π-bond (**b**)) toward the Cu atom in compounds ***2Si’*** and **2Ge’**. For each donor-acceptor interaction shown, the interaction energy is given in kcal mol^−1^.

In addition to this Cu→*E* (*E* = Si, Ge) donation, for compounds ***2Si’*** and **2Ge’**, second-order perturbation analysis of the Natural Bond Orbitals (NBOs) detected (*E*–C)→Cu and (C=C)→Cu donation from orbitals of the tetrel-bound phenyl group into a Cu atom’s NBO, which is dominated by 4s character. These interactions are represented in Figure 8. Both compounds benefit (by ca. 2 kcal mol^−1^) from electron donation out of the NBO representative of the *E*–C_ipso_ σ-bond toward Cu (interactions shown in column (a)), and a second contribution of electron donation toward Cu originates from one of the phenyl group’s 1,2-C=C bond NBOs (shown in column (b)). The latter is more pronounced for compound ***2Si’*** (2.4 kcal mol^−1^) than for compound **2Ge’** (1.1 kcal mol^−1^). These additional bonding interactions provide an explanation for the pronounced bending of the Ge-bound phenyl group in **2Ge’** to the Cu atom (as observed in the X-ray structure analysis), the offset of the BCP of the *E*–Cu bond in compounds **2*E*’** toward the *E*-bound phenyl group (which is particularly pronounced for ***2Si’***), the slightly enhanced WBI and DI observed (as well as the pronounced ellipticity), and the electron localization pointed out with the red arrows in the ELF graphics in Figure 6. For Pd-Ge-compound **XIII** [16] and its silicon analog, the authors have also identified such a combination of σ-donation from the d^10^-ML_3_ site to the tetrel and back-donation from, in particular, the Ge–C_ipso_ or Si–C_ipso_ σ-bond to the Pd atom. Of note, for these compounds, the authors report back-donation into Pd-localized NBOs with predominant p-character, whereas for ***2Si’*** and **2Ge’**, we detected back-donation into Cu-localized NBOs with predominant s-character.

Last but not least, second-order perturbation analysis of the NBOs in ***2Si’*** and **2Ge’** provides an indication as to why isomer **2Si** is more stable than ***2Si’***, but for germanium, **2Ge’** is more stable than ***2Ge***. Release of one of the three Cu–N bonds in isomer **2*E*** can in principle be compensated by remote N⋯*E* coordination of this N atom’s lone pair in isomer **2*E*’**. In the optimized molecular structures of ***2Si’*** and **2Ge’**, the N⋯*E* distances are 2.86 and 2.55 Å, respectively. This already hints at stronger N⋯*E* coordination for *E* = Ge. For both compounds, second-order perturbation analysis of the NBOs detected two donor-acceptor interactions from the N-atom´s lone pair into *E*-located NBOs. The associated interaction energies for the germanium compound **2Ge’** (7.5 and 6.8 kcal mol^−1^) are noticeably more pronounced than for its lighter congener ***2Si’*** (1.7 and 1.5 kcal mol^−1^).

## 3. Materials and Methods

### 3.1. General Considerations

Starting materials, **1Ge** [22] and CuCl [33], were available from previous studies. Diethyl ether was dried using an MBraun SPS-800 setup (MBraun, Garching, Germany). CDCl_3_ (Deutero, Kastellaun, Germany, 99.8%) was stored over activated molecular sieves (3 Å) for at least 7 days and used without further purification. All reactions were carried out under an atmosphere of dry argon, utilizing standard Schlenk techniques. (The starting material **1Ge** and the Cu complexes reported in this paper are sensitive toward hydrolysis, and the Cu(I) complexes are sensitive toward oxidation.) Solution NMR spectra (^1^H, ^13^C) (cf. Figures S1 and S2 in the Supporting Information) were recorded on a Bruker Avance III 500 MHz spectrometer (Bruker Biospin, Rheinstetten/Karlsruhe, Germany). The chemical shifts are reported relative to Me_4_Si (0 ppm) as an internal reference. ^1^H and ^13^C NMR signals were assigned according to the shifts of corresponding ^1^H or ^13^C NMR signals in related compounds **1Ge** [22] and **2Si** [14]. Elemental analyses were performed on an Vario MICRO cube (Elementar, Langenselbold, Germany). For single-crystal X-ray diffraction analyses, crystals were selected under inert oil and mounted on a glass capillary (which was coated with silicone grease). Diffraction data were collected on a Stoe IPDS-2 diffractometer (STOE, Darmstadt, Germany) using Mo Kα-radiation. Data integration and absorption correction were performed with the STOE software XArea and XShape, respectively. The structures were solved using SHELXT [34] and refined with the full-matrix least-squares methods of *F*^2^ against all reflections with SHELXL-2018/3 [35,36]. All non-hydrogen atoms were anisotropically refined; hydrogen atoms were isotropically refined in an idealized position (riding model). For details of data collection and refinement, see Appendix B, Table A1. Graphics of molecular structures were generated with ORTEP-3 [37,38] and POV-Ray 3.7 [39].

The geometry optimizations were carried out with ORCA 5.0.3 [40] using the unrestricted PBE0 functional with a relativistically recontracted Karlsruhe basis set ZORA-def2-TZVPP [41,42] (for H, C, N, O, Cl, Si, Ge, Cu), the scalar relativistic ZORA Hamiltonian [43,44], atom-pairwise dispersion correction with the Becke-Johnson damping scheme (D3BJ) [45,46] and COSMO solvation (CHCl_3_, ε = 4.8, rsolv = 3.17). VeryTightSCF and slowconv options were applied, with a radial integration accuracy of 10 for Si, Ge, and Cu for all calculations. Calculations were started from the molecular structures obtained by single-crystal X-ray diffraction analysis, and isomers were created by modifying these structures. Numerical frequency calculations were performed to prove convergence at the local minimum after geometry optimization and to obtain the Gibbs free energy (293.15 K). On the final structures, single-point calculations were performed with an unrestricted B2T-PLYP functional with relativistically recontracted Karlsruhe basis sets ZORA-def2-TZVPP [41,42] (for H, C, N, O, Cl, Si, Ge, Cu) and utilizing the AutoAux generation procedure [47], the scalar relativistic ZORA Hamiltonian [43,44], atom-pairwise dispersion correction with the Becke-Johnson damping scheme (D3BJ) [45,46] and COSMO solvation (CHCl_3_, ε = 4.8, rsolv = 3.17). VeryTightSCF and slowconv options were applied, and DEFGRID3 was used with a radial integration accuracy of 10 for Si, Ge, and Cu for all calculations. NBO and NLMO calculations were performed with the NBO7 package [48]. NCI, ELF, QTAIM, Wiberg Bond Index, and DI calculations were carried out by using MultiWFN [49] at the same level of theory as used for NBO calculations. NLMO and ELF graphics were generated using ChemCraft [50]. The NCI results (visualized at a cutoff value of 0.45) were depicted with VMD [51].

### 3.2. Synthesis and Characterization

Compound **2Ge’** ((pyO)PhGe(μ^2^-pyO)_2_CuCl, C_21_H_17_ClCuGeN_3_O_3_). A Schlenk flask was charged with a magnetic stirring bar, PhGe(pyO)_3_ (compound **1Ge**, 80 mg, 0.185 mmol) and CuCl (16 mg, 0.162 mmol), evacuated and set under an Ar atmosphere prior to adding CDCl_3_ (0.65 mL). The resultant dispersion was stirred at room temperature to afford a clear solution (almost colorless, slightly light-blue) within less than 2 min. After 15 min the solution was layered with diethyl ether (1 mL) and stored at room temperature. In the course of 2 days, colorless crystals of **2Ge’** formed, from which the supernatant was removed with a syringe (and transferred into another Schlenk tube under Ar atmosphere), and the crystals were dried in a vacuum. Yield: 50 mg (0.094 mmol, 58%). Elemental analysis for C_21_H_17_ClCuGeN_3_O_3_ (549.00 g·mol^−1^): C, 47.50%; H, 3.23%; N, 7.91%; found C, 45.57%; H, 3.40%; N, 7.66%. The data were found to correspond to the composition C_21_H_17_ClCuGeN_3_O_3_·H_2_O (530.96 g·mol^−1^): C, 45.94%; H, 3.49%; N, 7.65%. Traces of moisture may have entered the sample during preparation for elemental analysis, but the lower C and higher H content may also arise from solvent residues (CDCl_3_ and Et_2_O) or a combination of both effects. ^1^H NMR (CDCl_3_): *δ* (ppm) 6.8–7.0 (2s, broad, 6H), 7.4–7.5 (s, broad, 2H), 7.55–7.65 (s, broad, 3H), 7.65–7.75 (s, broad, 3H), 8.1–8.3 (s, broad, 3H); ^13^C{^1^H} NMR (CDCl_3_): *δ* (ppm) 162.5 (C^2^), 147.0 (C^6^), 141.2 (C^4^), 133.9 (Ph-o), 132.9 (Ph-p), 129.2 (Ph-m), 117.3 (C^5^), 113.8 (C^3^) (the superscript numbers indicate the position in the pyridine-2-olate heterocycle). The signal of the ipso-C atom was not detected.

From the supernatant, which turned deep blue upon transfer into another Schlenk tube, dark blue crystals of **3Ge** formed within 3 days. These crystals were isolated by decantation and dried in a vacuum. Yield: 6 mg (0.010 mmol). Elemental analysis for C_26_H_21_ClCuGeN_4_O_4_ (625.05 g·mol^−1^): C, 49.96%; H, 3.39%; N, 8.96%; found C, 50.02%; H, 3.13%; N, 9.03%. As compound **3Ge** forms upon access to air, we concluded that **3Ge** can be prepared in an analogous manner to compound **3Si** [14] (using CuCl, dry air, and, instead of excess **1Si**, excess **1Ge**). Because of the small amount of **1Ge** and **2Ge’** available, we did not aim at optimizing the synthesis of **3Ge**. 

## 4. Conclusions

The reaction of the pyridine-2-olato functionalized germane PhGe(pyO)_3_ (**1Ge**) and CuCl afforded the heteronuclear complex **2Ge,’** which features two bridging pyO ligands and one exclusively Ge-bound pyO ligand. This unexpected coordination mode, which is in contrast to the (μ^2^-pyO)_3_ motif in the silicon congener **2Si** [14], can be attributed to enhanced (κ^2^-pyO) coordination at Ge. Compound **2Ge’** is highly sensitive toward air (both oxygen and moisture), and access to traces of air gave rise to the formation of the heteronuclear Cu(II) complex **3Ge**. A computational comparison of the Cu–Si and Cu–Ge bonds in couples of congeners **2*E***, **2*E*’**, and **3*E*** (*E* = Si, Ge) revealed stronger interactions for the Ge compounds. Whereas in **2*E*** and **3*E*** the nature of these weak bonds is Cu→*E* σ-donation, in **2*E*’** the bond comprises an additional contribution of σ back-donation from bond electron pairs of the *E*-bound phenyl group toward Cu. Such a bonding mode has previously been described for phosphine-bridged Pd(0)–*E* complexes [16]. As in these compounds, the coordination of the d^10^ site at *E* became relevant for the activation of further bonds at *E* (*E*–F-bond activation for cross-coupling reactions), related Cu(I)-coordination motifs at *E* may become of interest for catalysis as well. From the academic point of view, our new complexes **2Ge’** and **3Ge** represent the first crystallographically characterized examples of compounds with rather short Cu→Ge coordination (below 3 Å), and the dinuclear core in **2Ge’** delivers the first crystallographic evidence of intense (*E*–C_ipso_)→Cu coordination. Thus, these compounds contribute to the portfolio of compounds with d-block→p-block coordination, a topic of current interest [52]. With germanium as the p-block Z-type ligand site, this study adds to our understanding of the so-called tetrel bonds [53,54].

## Figures and Tables

**Table 1:** Values of *sign*(*λ*_2_(**r_b_**))*ρ*(**r_b_**) at the Cu–*E* bond critical points (BCPs) of compounds **2Si**, ***2Ge***, ***2Si’***, **2Ge’**, **3Si,** and **3Ge**.

	2*E*	2*E*’ ^1^	3*E*
*E* = Si	−0.01408	−0.03303	−0.01896
*E* = Ge	−0.02325	−0.03113	−0.02026

^1^ These BCPs are not located on the Cu–E-axis, the bond paths are strongly deformed, and the BCPs are offset toward the phenyl group’s ipso-C atom (cf. Figure S11).

**Table 2:** Selected features of (3, −1) critical points in compounds **2Si**, ***2Si’***, ***2Ge***, **2Ge’**, **3Si,** and **3Ge**, which relate to the Cu–*E* (*E* = Si, Ge) bond or, in the case of **2*E*’** (*E* = Ge, Si; for ***2Si’*** in particular), to the Cu,*E*,C_ipso_ multi-center bond (electron density *ρ*(**r_b_**) in au, Laplacian of the electron density ∇^2^*ρ*(**r_b_**) in au, Lagrangian kinetic energy density *G*(**r_b_**) in au, potential energy density *V*(**r_b_**) in au, ratio |*V*(**r_b_**)|/*G*(**r_b_**) in au, electron energy density *H*(**r_b_**) in au, ellipticity of the electron density *ε*, Electron Localization Function (ELF) in au), Wiberg Bond Index (WBI), and total Delocalization Index (DI) of the Cu–*E* (*E* = Si, Ge) bond.

	*ρ*(r_b_)	∇^2^*ρ*(r_b_)	*G*(r_b_)	*V*(r_b_)	|*V*(r_b_)|/*G*(r_b_)	*H*(r_b_)	*ε*	ELF	WBI	DI
**2Si**	0.0141	0.0209	0.0068	−0.0083	1.229	−0.0016	0.356	0.107	0.104	0.015
***2Ge***	0.0233	0.0177	0.0090	−0.0136	1.508	−0.0046	0.015	0.267	0.212	0.117
***2Si’***	0.0330	0.0826	0.0232	−0.0257	1.109	−0.0025	1.257	0.151	0.242	0.238
**2Ge’**	0.0311	0.0450	0.0163	−0.0213	1.308	−0.0050	2.162	0.228	0.267	0.138
**3Si**	0.0190	0.0245	0.0091	−0.0120	1.324	−0.0029	0.030	0.154	0.141	0.028
**3Ge**	0.0203	0.0299	0.0103	−0.0131	1.272	−0.0028	0.008	0.151	0.155	0.064

**Table 3:** Natural Charges (NCs) of Cu- and Si- or Ge-atoms (*E*) and contributions to the NLMO of the Cu–*E* σ-bond of compounds **2Si**, ***2Si’***, ***2Ge***, **2Ge’**, **3Si,** and **3Ge**.

	NC(Cu)	NC(*E*)	NLMO σ-Cu–*E*	Cu+*E*
**2Si**	0.86	2.25	97.9% Cu, 0.9% Si	98.8%
***2Ge***	0.89	2.14	94.9% Cu, 3.2% Ge	98.1%
***2Si’***	0.85	2.22	95.5% Cu, 1.7% Si	97.2%
**2Ge’**	0.86	2.11	94.9% Cu, 2.7% Ge	97.6%
**3Si**	1.32	2.14	98.4% Cu, 0.9% Si	99.3%
(α-spin)			(98.7% Cu, 0.7% Si)	
(β-spin)			(98.2% Cu, 1.1% Si)	
**3Ge**	1.33	2.13	98.3% Cu, 1.0% Ge	99.3%
(α-spin)			98.6% Cu, 0.8% Ge	
(β-spin)			97.9% Cu, 1.3% Ge	

## Data Availability

CCDC 2269071 (**2Ge’**) and 2269072 (**3Ge**) contain the supplementary crystal data for this article. These data can be obtained free of charge from the Cambridge Crystallographic Data Centre via https://www.ccdc.cam.ac.uk/structures/ (accessed on 11 June 2023).

## Supplementary Materials

The following supporting information can be downloaded at: https://www.mdpi.com/article/10.3390/molecules28145442/s1. Crystallographic data for the compounds reported in this paper (in CIF format) and a document containing the following: NMR spectra (^1^H and ^13^C{^1^H}) of **2Ge’** (Figures S1 and S2), comparison of corresponding NMR spectra of **2Ge’** and **2Si** (Figures S3 and S4); graphics of optimized molecular structures and total energies (Figures S5–S10) as well as atomic coordinates (Tables S1–S6) of compounds ***2Ge***, **2Ge’**, **3Ge**, **2Si**, ***2Si’*** and **3Si**; graphics of the bond paths and bond critical points identified in the optimized molecular structures of ***2Si’*** and **2Ge’** (Figure S11); graphical representations of the α- and β-spin contributions to the NLMOs representative of the Cu→Si and Cu→Ge σ-interactions in **3Si** and **3Ge**, respectively (Figure S12), 1D plots of the Laplacian of electron density along the Cu–*E* (*E* = tetrel) bond paths of compounds ***2Ge***, **2Ge’**, **3Ge**, **2Si**, ***2Si’*** and **3Si** (Figure S13).

## Sample Availability

The compounds reported in this paper were prepared in small quantities, sufficient for characterization within the context of this study. Thus, no samples are available from the authors.

## Appendix A

This refers to a search in the Cambridge Structure Database using ConQuest version 2023.1.0 [17]. The search for structures with potential Cu→Ge dative bonding was performed for all compounds available which exhibit a Cu⋯Ge interatomic distance in the range 2.4–3.5 Å (the latter value was chosen to yield hits which feature Cu⋯Ge distances well below the sum of the van der Waals radii of 4.1 Å), any bond type and also absence of a bond were allowed, and compounds with Ge–Ge bonds were excluded. The search yielded 71 hits, which were inspected individually to exclude examples which can be interpreted as covalently bound Cu-germyl-compounds, as germylene complexes of Cu or as forced short contact because of rigid bridging ligands (e.g., Cu and Ge atoms as constituents within small rings). Furthermore, the molecular structures were inspected for geometric response of the Cu- and/or Ge-coordination sphere(s) to the Cu⋯Ge close proximity (e.g., planarization of otherwise tetrahedral faces of the Ge coordination sphere, bending of otherwise linear Cu(I) coordination spheres, pointing of the vacant site of T-shaped Cu coordination spheres or of the face of a trigonal planar Cu coordination sphere toward Ge etc.).

## Appendix B

**Table A1:** Crystallographic data from data collection and refinement for **2Ge’** and **3Ge**.

Parameter	2Ge’	3Ge
Formula	C_21_H_17_ClCuGeN_3_O_3_	C_26_H_21_ClCuGeN_4_O_4_
*M*_r_	530.96	625.05
*T*(K)	180(2)	180(2)
*λ*(Å)	0.71073	0.71073
Crystal system	monoclinic	orthorhombic
Space group	*P*2_1_/*n*	*Pbca*
*a*(Å)	14.4369(2)	15.7300(2)
*b*(Å)	8.7499(1)	18.2546(4)
*c*(Å)	16.2903(3)	17.1794(3)
*β*(°)	92.555(1)	90
*V*(Å^3^)	2055.77(5)	4932.98(15)
*Z*	4	8
*ρ*_calc_(g·cm^−1^)	1.72	1.68
*μ*_MoKα_ (mm^−1^)	2.7	2.2
*F(000)*	1064	2520
*θ*_max_(°), *R*_int_	28.0, 0.0417	28.0, 0.0720
Completeness	100%	100%
Reflns collected	49,966	72,136
Reflns unique	4971	5957
Restraints	0	0
Parameters	272	335
GoF	1.097	1.085
*R*1, *wR*2 [*I* > 2*σ*(*I*)]	0.0263, 0.0651	0.0356, 0.0761
*R*1, *wR*2 (all data)	0.0309, 0.0671	0.0509, 0.0817
Largest peak/hole (e·Å^−3^)	0.38, −0.45	0.48, −0.52
