# Supplementary material for: Ge–Cu-Complexes Ph(pyO)Ge(μ2-pyO)2CuCl and PhGe(μ2-pyO)4CuCl—Representatives of Cu(I)→Ge(IV) and Cu(II)→Ge(IV) Dative Bond Systems
Source: Molecules. 2023 Jul 16;28(14):5442. doi: 10.3390/molecules28145442 (PMC10385301; doi:10.3390/molecules28145442)
Supplement: Supplementary file 1 [file molecules-28-05442-s001.zip › Supporting_information_2474623.pdf]

Ge–Cu-Complexes $Ph(pyO)Ge(\mu^2\text{-}pyO)_2CuCl$ and $PhGe(\mu^2\text{-}pyO)_4CuCl$ – Representatives of Cu(I)→Ge(IV) and Cu(II)→Ge(IV) Dative Bond Systems

Jörg Wagler[1], Robert Gericke[2]*

[1] Institut für Anorganische Chemie, TU Bergakademie Freiberg, 09596 Freiberg, Germany

[2] Institute of Resource Ecology, Helmholtz-Zentrum Dresden-Rossendorf eV, 01328 Dresden, Germany; gericker.chemie@gmail.com

Supporting Information:

Content:

- NMR spectra (^1H and $^{13}C\{^1H\}$) of **2Ge'** and comparison with NMR spectra (^1H and $^{13}C\{^1H\}$) of **2Si**
- Molecular graphics, total energies and atomic coordinates of the optimized molecular structures of compounds ***2Ge***, **2Ge'**, **3Ge**, **2Si**, ***2Si'***, **3Si**
- Bond paths with Bond Critical Points identified for the optimized molecular structures of ***2Si'*** and **2Ge'**
- α- and β-spin contributions to the NLMOs representative of the Cu→Si and Cu→Ge σ-interactions in **3Si** and **3Ge**, respectively
- 1D plots of the Laplacian of electron density along the Cu–*E* (*E* = Si, Ge) bond path in ***2Ge***, **2Ge'**, **3Ge**, **2Si**, ***2Si'***, **3Si**

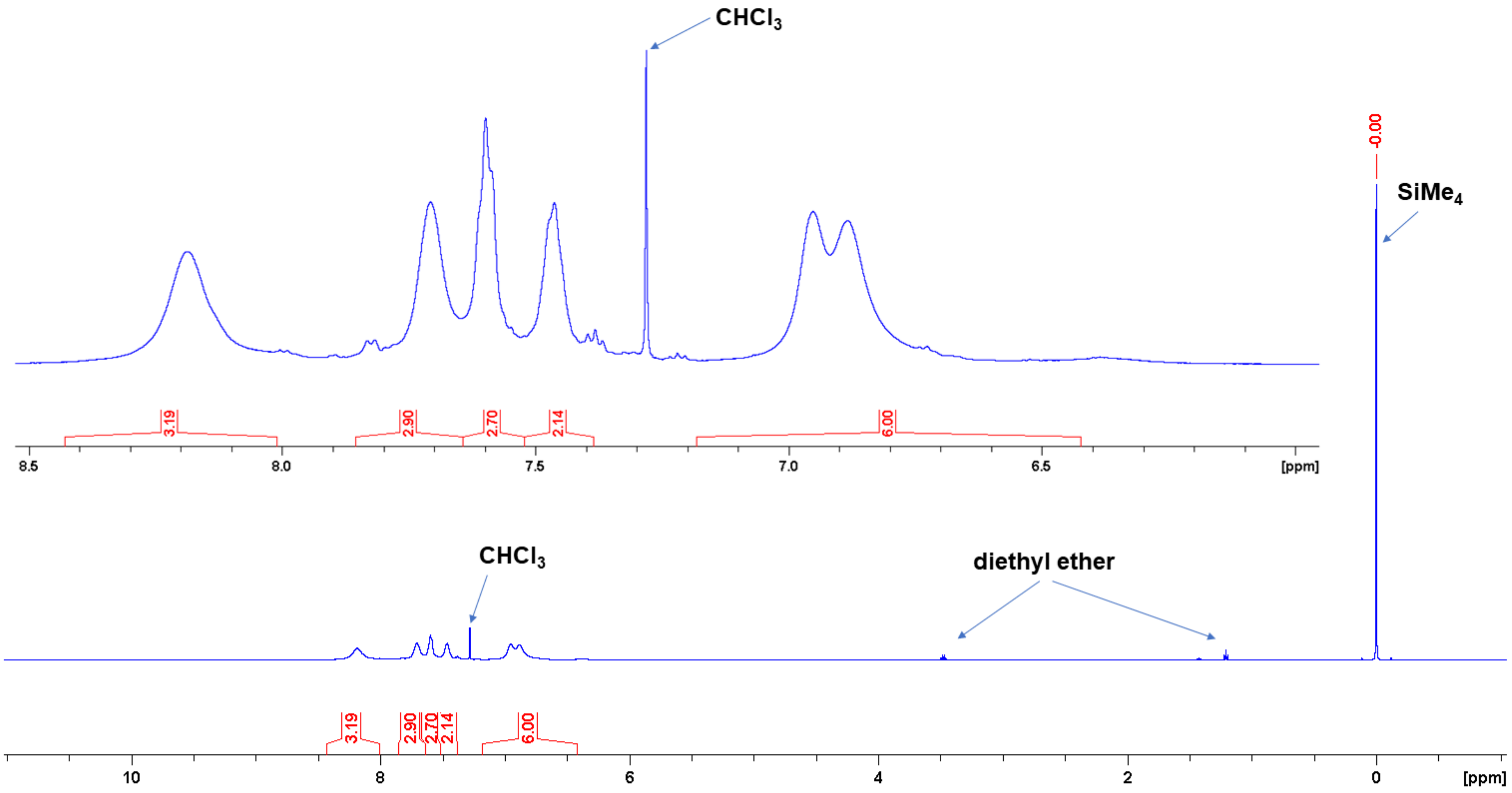

Figure S1. ^{1}H NMR spectrum of **2Ge'** in $CDCl_3$ (full spectrum and magnified inset of the group of signals).

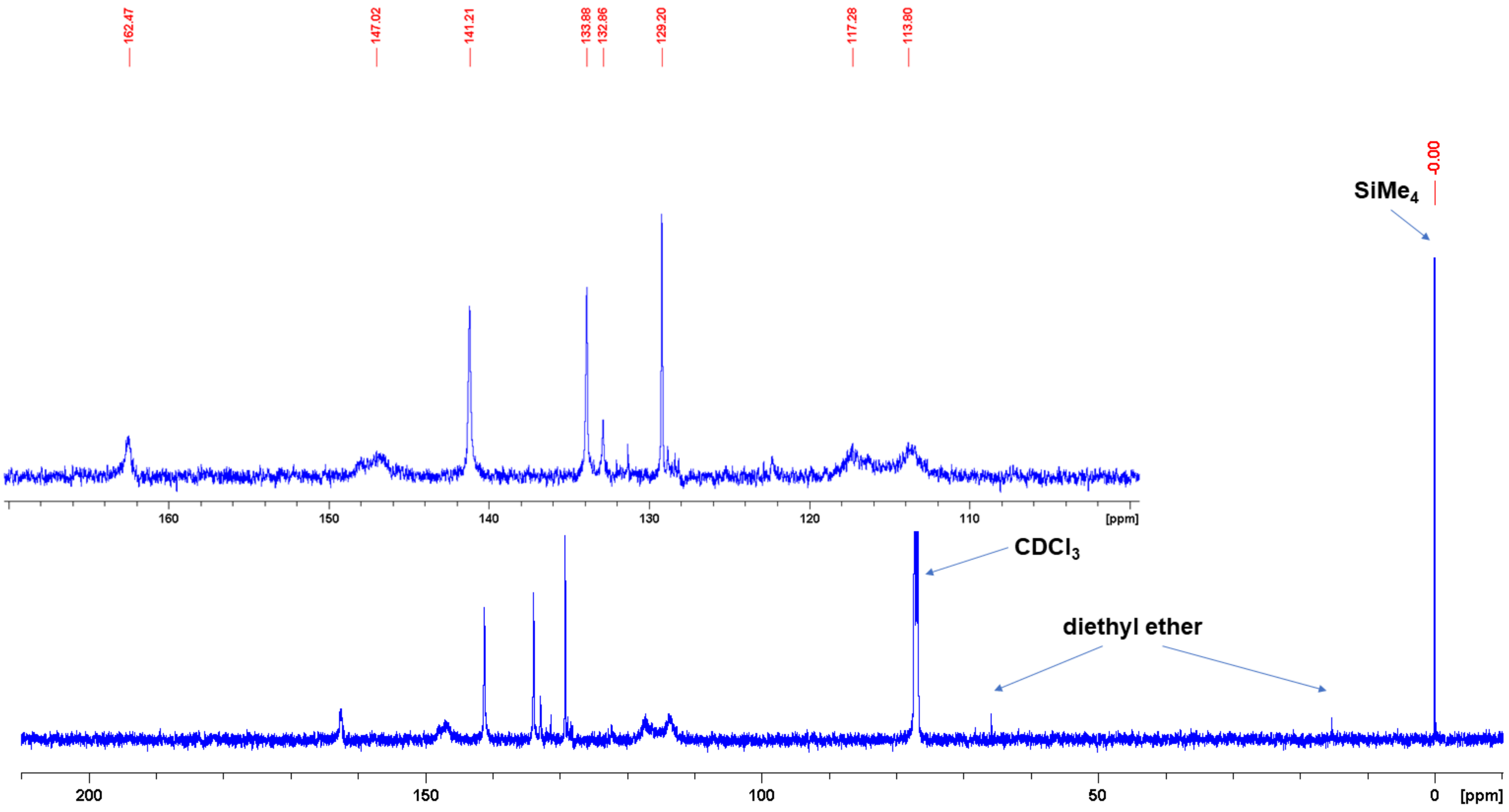

Figure S2. $^{13}C\{^{1}H\}$ NMR spectrum of **2Ge'** in $CDCl_3$ (full spectrum and magnified inset of the aryl part of signals).

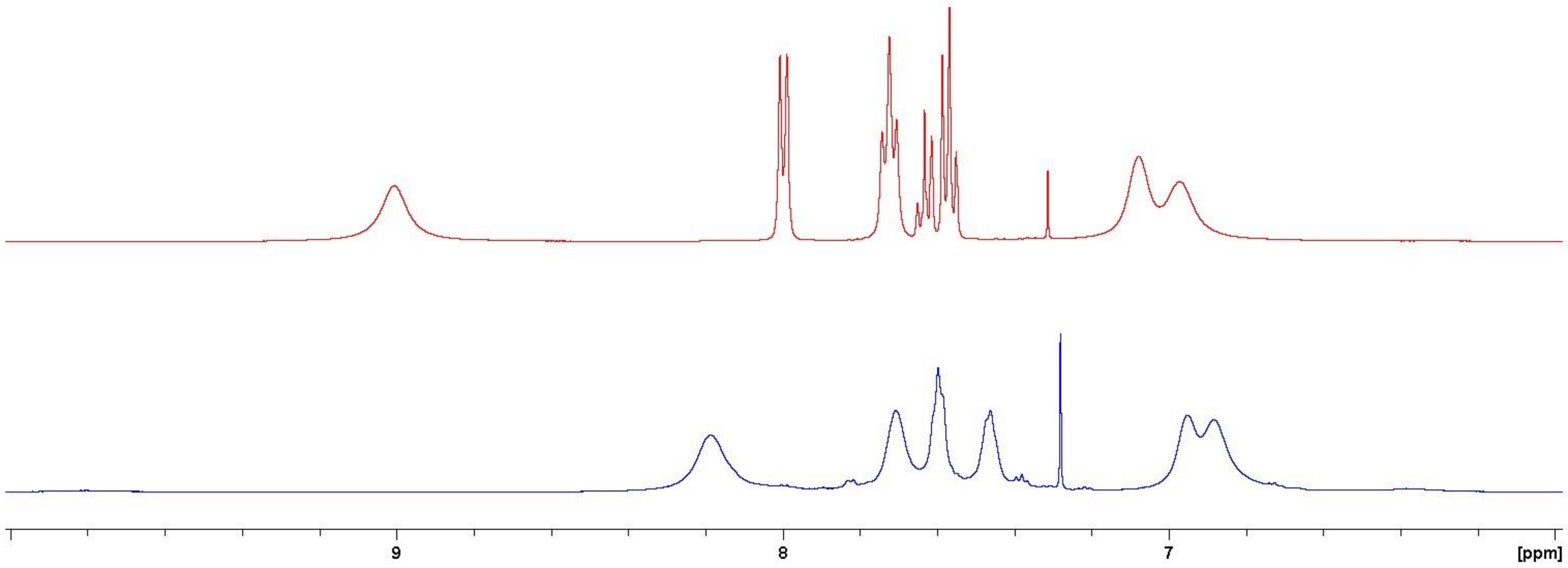

Figure S3. Comparison of the ^{1}H NMR spectra of **2Ge’** (bottom, blue trace) and **2Si** (top, red trace) in $CDCl_3$.

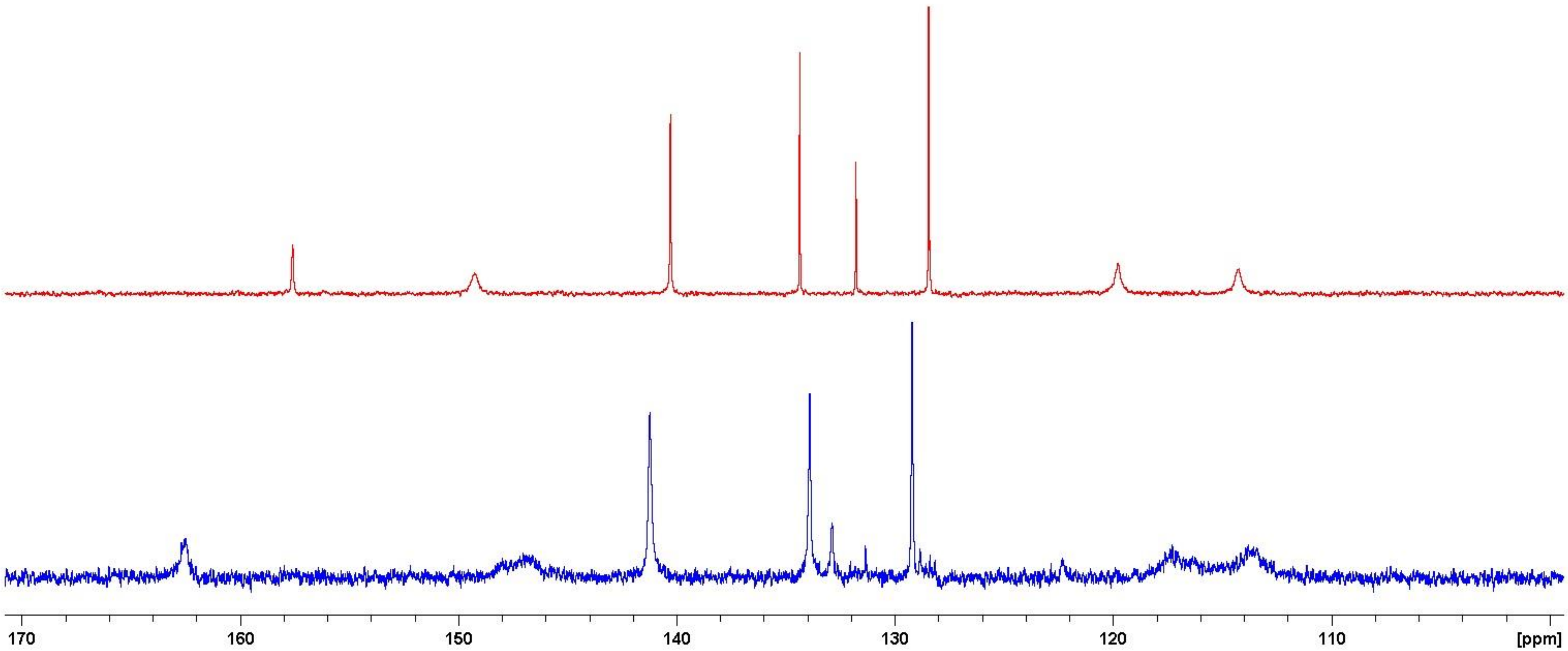

Figure S4. Comparison of the $^{13}C\{^1H\}$ NMR spectra of **2Ge'** (bottom, blue trace) and **2Si** (top, red trace) in $CDCl_3$.

Atomic coordinates and total energies:

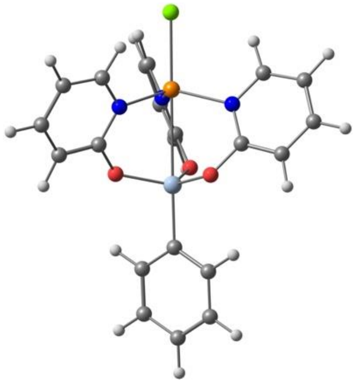

Figure S5. Optimized molecular structure of ***2Ge***.

PBE0:

final single point energy: -5436.6039141624 a.u.

final Gibbs free energy: -5436.31142998 a.u.

B2T-PLYP:

final single point energy: -5436.71095987033 a.u.

Table S1. Atomic coordinates for optimized structure of ***2Ge***.

Cu	0.14897521388847	-0.13313745199636	-0.22502963224329
Cl	0.20793180558470	-0.23309435163438	2.13467976201013
Ge	0.07514223677273	0.00508556326343	-3.14105302123336
O	1.71470125425097	-0.65616080639645	-2.83607052115161
O	-0.21417249588846	1.70570866051641	-2.65025699528070
O	-1.24670394267924	-1.15591848861858	-2.78365892828327
N	2.10165170080671	0.05925176547041	-0.68653122650424
N	-1.06047532523758	1.44578505787519	-0.53278320014212
N	-0.64642030994669	-1.93124030603041	-0.70449314189164
C	2.57126534008522	-0.30580067110235	-1.87215808912223
C	3.93399053497252	-0.36160387802063	-2.15792979462319
H	4.25620761020072	-0.67478403302194	-3.14120776043599
C	4.82372423360078	-0.00511491461070	-1.16797387238837
H	5.88922096799728	-0.03571367620535	-1.36067778723004
C	4.33810813301369	0.39734561905028	0.07203662325117
H	5.00241581417974	0.68803042740613	0.87411729636212
C	2.97612122985690	0.41111569088781	0.26856092286227
H	2.53099136951369	0.68276122232872	1.21789432220104
C	-1.00995353790652	2.13461815115919	-1.66458737362560
C	-1.73657435287504	3.30530812705922	-1.86835812692064
H	-1.64847642819682	3.82735267566521	-2.81100363960837
C	-2.55265243782967	3.75188394704788	-0.85123403590007
H	-3.13349393616229	4.65712103248869	-0.97997804666271
C	-2.62340306254598	3.02588741113718	0.33318613820796
H	-3.25378858769780	3.34041755312488	1.15354186365503
C	-1.86133469696457	1.88544323401691	0.44969219602642
H	-1.84750770672684	1.28808419223332	1.35315433658331

C	-1.27257750835645	-2.11913716314582	-1.85801391108125
C	-1.98623142739880	-3.28097133269966	-2.14776931966035
H	-2.48171647202917	-3.37313588366122	-3.10422860906327
C	-2.02965457107942	-4.27496238347453	-1.19467384400303
H	-2.57708330703952	-5.18921251769452	-1.38979032057643
C	-1.36132261852518	-4.09162741508309	0.01162702610363
H	-1.36879652653703	-4.84812510953027	0.78423787399029
C	-0.68724017149509	-2.90894872715058	0.21365652759508
H	-0.17138450798138	-2.68846030309428	1.14020701461787
C	-0.00018326732252	0.11120735761170	-5.05259565767649
C	-0.55216715804003	1.22328138974099	-5.68076407822839
H	-0.91081189182074	2.05842007946709	-5.09203617360706
C	-0.64359360226921	1.26464275010779	-7.06514194374849
H	-1.07417042270415	2.13228727945164	-7.55045731216074
C	-0.18617087556342	0.19743717282258	-7.82387960536344
H	-0.25914199080041	0.23081554558997	-8.90440686072140
C	0.36569495309758	-0.91384088339331	-7.20042085231548
H	0.72362885238249	-1.74773393672768	-7.79215283879104
C	0.45842115744200	-0.95902834519622	-5.81787793680500
H	0.89139493997379	-1.83051868203421	-5.34005996641692

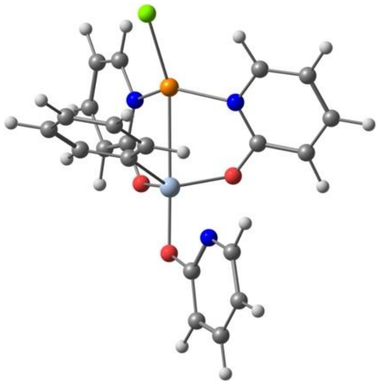

Figure S6. Optimized molecular structure of **2Ge'**.

PBE0:

final single point energy: -5436.61267096513 a.u.

final Gibbs free energy: -5436.32015284 a.u.

B2T-PLYP:

final single point energy: -5436.71581360334 a.u.

Table S2. Atomic coordinates for optimized structure of **2Ge'**.

Cu	-0.12260782931954	-0.01269115738970	-0.07092791898456
Cl	-0.34854218815825	-0.96485157095888	1.97925968160931
Ge	-0.24267715821137	-0.03687362214155	-2.82073839617267
O	-1.49485977485086	1.25685797253810	-2.68322423561242
O	1.30086060928349	0.87238173029083	-2.59411709547303
O	-0.36639898823002	-0.01687417366246	-4.63480898867218
N	-1.57009322432262	1.29609571011305	-0.37694172622844
N	1.79010223665460	-0.08614239817844	-0.57676030675560
N	1.28832828675063	-1.48430417163475	-4.25431731152655
C	-1.99770114039682	1.74327449583953	-1.55949831168023
C	-2.98867075666000	2.72307635032990	-1.66525155348009
H	-3.29589978306954	3.04741684859684	-2.64970358823181
C	-3.53803925188093	3.23828538539943	-0.51502262641188
H	-4.30660890030913	3.99919358613746	-0.57555165989777
C	-3.09946282257074	2.76868793696158	0.72076890310875
H	-3.50865566185506	3.14155249169422	1.64921026153485
C	-2.12394712694078	1.80238114730138	0.74084447854837
H	-1.75045586048066	1.38298134747430	1.66720400761882
C	2.21170287893368	0.42655530917968	-1.73285731904358
C	3.56394501160947	0.51675320657645	-2.05422558066011
H	3.85380763885822	0.93797510531759	-3.00660394792764
C	4.48591531054699	0.04714993661118	-1.14321444712339
H	5.54397336803411	0.09903497643875	-1.36985983545217
C	4.04747916844754	-0.50247476896831	0.05787569295431
H	4.74169478515203	-0.88611426741792	0.79255976476687
C	2.69377257934278	-0.55014967722713	0.29933472839809
H	2.27827575134250	-0.95999737770290	1.21239614347586
C	0.53668649967244	-0.85319269207524	-5.14624781274998
C	0.67056112401303	-1.04044484575840	-6.52090760494311
H	0.03264781949841	-0.50316999125829	-7.20896073965181
C	1.63783473386116	-1.92735466187283	-6.94747235912899
H	1.77679175216266	-2.10265857134676	-8.00759330944653

C	2.43198408830587	-2.59467231744314	-6.01728840726655
H	3.19660228561674	-3.29381535711452	-6.32691989165660
C	2.21751780408627	-2.34078146295950	-4.67798061898650
H	2.80347835598950	-2.83314685034254	-3.90941685465990
C	-0.85529965968786	-1.64376455924259	-1.95923909736014
C	-0.00013085104900	-2.70007529899103	-1.64870972245148
H	1.05406944910481	-2.63284376295607	-1.87986301838435
C	-0.50472837084066	-3.83578067959401	-1.03895044917059
H	0.16307123563607	-4.65053386069458	-0.78778368554991
C	-1.85892648758688	-3.92828337459667	-0.74641143552033
H	-2.24835742872789	-4.81771879787473	-0.26635887231424
C	-2.71369442441002	-2.88319284832080	-1.06429849210222
H	-3.77039561938708	-2.95372943686992	-0.83836094379914
C	-2.21631548755057	-1.73762025477080	-1.66515777981739
H	-2.88971444840674	-0.92263670643581	-1.90075653372125

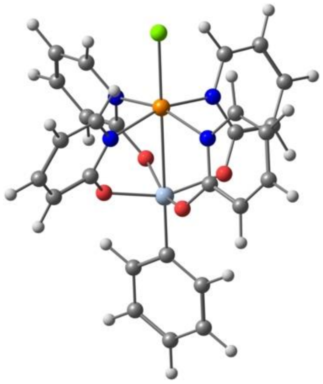

Figure S7. Optimized molecular structure of **3Ge**.

PBE0:

final single point energy: -5759.596763416248 a.u.

final Gibbs free energy: -5759.22280124 a.u.

Table S3. Atomic coordinates for optimized structure of **3Ge**.

Cu	-0.03114815450733	-0.01960511092841	0.01455741724332
Cl	-0.03477659046309	-0.05813223711715	2.42749456450320
Ge	-0.03098624353531	0.02293724757878	-2.85816550299053
O	0.74683824549580	1.67757427509364	-2.42034118725434
O	1.63177950706148	-0.75349131306483	-2.46230403735216
O	-0.81033849449399	-1.64380029295469	-2.47013978542499
O	-1.69416031664548	0.78679943770028	-2.44159580081500
N	-0.03017037264754	1.99120530928097	-0.28741444012854
N	1.97628715481149	-0.01595160770491	-0.31935127144190
N	-0.02850334189997	-2.02221115959725	-0.35012220464519
N	-2.03871975936565	-0.01903629561715	-0.32349082705192
C	0.37617228022299	2.48751087739226	-1.46215404696397
C	0.44574958070022	3.87193759785342	-1.66787612932299
H	0.78168724044828	4.23423310721692	-2.62976883184461
C	0.07871198007905	4.71949700647022	-0.65052817613292
H	0.12514733865889	5.79221345250637	-0.79708414541938
C	-0.36084433339472	4.19015542084490	0.56184303471372
H	-0.66375721951053	4.82402842427511	1.38351658987571
C	-0.39879440045919	2.82359101817322	0.69616701061365
H	-0.71296570670506	2.34076675214916	1.61343453061924
C	2.45540662522949	-0.39842534980222	-1.50887887903978
C	3.83644452992289	-0.46056822540252	-1.73738121083658
H	4.18481470494640	-0.77916492994316	-2.71035297401551
C	4.69900032972776	-0.11003172962216	-0.72667351755116
H	5.76944394854551	-0.15138030990135	-0.89040610551655
C	4.18743328194578	0.30556038838780	0.50155578709531
H	4.83321723276118	0.59493337320127	1.31882938776783
C	2.82294828903556	0.33666600266109	0.65788171998858
H	2.35356817816990	0.63176884554108	1.58839590215904
C	-0.43502366918363	-2.48289776460837	-1.53912616890491
C	-0.49848940212838	-3.86046130309740	-1.78840751112801
H	-0.83522986950681	-4.19399274536769	-2.76038942490347
C	-0.12357512657632	-4.73793865155199	-0.79959315123041

H	-0.16427159283855	-5.80571742714075	-0.98012088404486
C	0.31680801590588	-4.24509141759030	0.42778715148264
H	0.62577450289327	-4.90324209872345	1.22784847103454
C	0.34729728171224	-2.88326981498699	0.60555372571289
H	0.66127758413252	-2.42794940809210	1.53679688046938
C	-2.51797154103299	0.39510677501442	-1.50233656083440
C	-3.89861049433916	0.45004078425742	-1.73395918386552
H	-4.24687588741130	0.79448709710803	-2.69811498252983
C	-4.76101345538464	0.05888643684233	-0.73800561897498
H	-5.83123031354311	0.09308933747526	-0.90475779639329
C	-4.24920724987726	-0.38876794493575	0.47871915755588
H	-4.89463649805443	-0.71046560791842	1.28410290761075
C	-2.88496935124971	-0.41042413619365	0.63914430082735
H	-2.41551795873295	-0.73014134527007	1.56145313245407
C	-0.02632351885022	0.04427968075263	-4.77374585236896
C	1.13980938952807	0.35380687853913	-5.46684256386262
H	2.04951821346611	0.58602376985917	-4.92529121061558
C	1.14374857238001	0.36455518387034	-6.85440801204670
H	2.05435770539082	0.60679861992481	-7.38968358046748
C	-0.01633739150984	0.06350094678139	-7.55453056192222
H	-0.01232908285646	0.07056871592574	-8.63829121118066
C	-1.18148180258256	-0.24688097193338	-6.86695005878115
H	-2.08803093413758	-0.48227187859907	-7.41207244268312
C	-1.18749369005885	-0.25548026815433	-5.47936030661873
H	-2.10060717068899	-0.49679438785767	-4.94755658062210

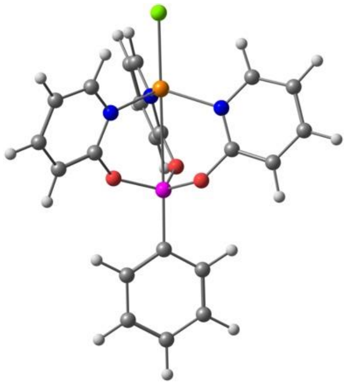

Figure S8. Optimized molecular structure of **2Si**.

PBE0:

final single point energy: -3616.13906064735 a.u.

final Gibbs free energy: -3615.84456878 a.u.

B2T-PLYP:

final single point energy: -3616.40582586454 a.u.

Table S4. Atomic coordinates for optimized structure of **2Si**.

Cu	0.08329990005294	-0.19456728683778	-0.04366898653440
Cl	0.16274542771764	-0.30086824928454	2.30270779810197
Si	0.00626142828641	-0.05286420408162	-3.16585760425200
O	1.51271044613200	-0.56868280862405	-2.76752754479302
O	-0.32034350457204	1.45586749451408	-2.62174315110727
O	-1.12069166104864	-1.17157023562995	-2.75198560295274
N	2.02305213447829	0.02617685076282	-0.61312047055423
N	-1.04116512530378	1.43489565456721	-0.44605923362648
N	-0.71174609606122	-1.95872069545556	-0.63305022675399
C	2.42760401243655	-0.21146683750584	-1.85199743432743
C	3.75842147140234	-0.13672511502752	-2.24596599162851
H	4.02139762887887	-0.34731415229772	-3.27339232256004
C	4.69805056080061	0.21246886281632	-1.29829325553025
H	5.74397843932592	0.28286247172792	-1.57112889913366
C	4.28479463996692	0.47266870142808	0.00332207888662
H	4.98938596672935	0.74828870743579	0.77612305175245
C	2.94375386365960	0.36617736195309	0.30033502857422
H	2.55670774918400	0.53503917115504	1.29868556449337
C	-1.04829888405423	2.00906743857314	-1.63946612349692
C	-1.74990308558150	3.17549073325164	-1.91995659622297
H	-1.71029167322172	3.59401347050373	-2.91616966577910
C	-2.47744563815355	3.75685076281443	-0.90225392203272
H	-3.03649455676600	4.66641863391849	-1.08502286392533
C	-2.48270386425794	3.16073468671928	0.35360118286110
H	-3.03790568704495	3.58512735981491	1.17900602193211
C	-1.75310707081588	2.00678107146807	0.53640820997382
H	-1.69803025990557	1.50188150866441	1.49395700759761
C	-1.19492555928137	-2.16334770603818	-1.84958628310024
C	-1.80600494053866	-3.35078642943455	-2.23640529234804
H	-2.18243999076768	-3.44938924756785	-3.24522776082374
C	-1.90703513805730	-4.36419666794683	-1.30655590934398

H	-2.37759560522504	-5.30236969782269	-1.57431108503574
C	-1.39731249875291	-4.16422555551064	-0.02850427371384
H	-1.45735651799753	-4.93180055597748	0.73101263262834
C	-0.81169563435773	-2.95234587246195	0.26322675990007
H	-0.41125457491560	-2.72405743891643	1.24434689173873
C	-0.04683975907486	0.06185536315860	-5.00161814855328
C	-0.32112868285644	1.26307125412649	-5.65568323555675
H	-0.51413482896244	2.15853807328645	-5.07689860987814
C	-0.34936064596530	1.32556649340799	-7.04176268127226
H	-0.56546248263265	2.26559397371983	-7.53560378804246
C	-0.10115792033720	0.18626417197843	-7.79269633385215
H	-0.12295728388012	0.23451301417026	-8.87508943817835
C	0.17418458385531	-1.01734408252244	-7.15645940406871
H	0.36738236817745	-1.90912743271786	-7.74083710007233
C	0.19987102493022	-1.07779494798569	-5.77248014940249
H	0.41357173237540	-2.02562742128933	-5.28901535998671

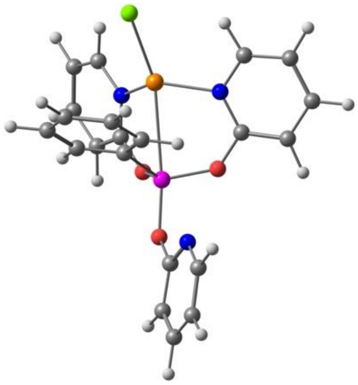

Figure S9. Optimized molecular structure of ***2Si'***.

PBE0:

final single point energy: -3616.14039179145 a.u.

final Gibbs free energy: -3615.84585217 a.u.

B2T-PLYP:

final single point energy: -3616.40416174907 a.u.

Table S5. Atomic coordinates for optimized structure of ***2Si'***.

Cu	-0.07490057266624	-0.04438281048499	0.05708187803364
Cl	-0.49176989881028	-1.00394035607315	2.07586154166559
Si	0.03058963676231	0.06158922228224	-2.76574605527622
O	-0.94994630659078	1.37919991320759	-2.55337862995076
O	1.60642171299714	0.47136945665679	-2.49225485493856
O	-0.07966748787537	0.02677097528729	-4.42137757523515
N	-1.50600060816862	1.26564724206907	-0.32813684944194
N	1.87765455359874	0.01908133592351	-0.26031427575876
N	1.33677097954487	-1.75246128830680	-4.55029564710207
C	-1.72450256619650	1.76706361121977	-1.53991464672511
C	-2.73930182107592	2.68312722031236	-1.80081168474511
H	-2.86937642293779	3.05374511177945	-2.80800016213297
C	-3.55128527542551	3.07486814572961	-0.75859171730072
H	-4.35253066435100	3.78255592950942	-0.93312711683120
C	-3.33539170321984	2.54683859329464	0.51010307235623
H	-3.95437044246753	2.82348160923175	1.35233450984796
C	-2.30709317138863	1.64859337456818	0.67905712997647
H	-2.09185054544712	1.18759975443739	1.63580110611595
C	2.41386021009878	0.26776765943260	-1.45100504098821
C	3.78806216587514	0.32948186464450	-1.65788950532280
H	4.16381844924997	0.53192203560792	-2.65105437356492
C	4.62019544155147	0.12153465487235	-0.57898230195756
H	5.69471680167904	0.16225785173740	-0.70904969913671
C	4.06742304567594	-0.14578370558194	0.66921340213155
H	4.68669635351331	-0.31869406452900	1.53845603712324
C	2.69698117377522	-0.19102710514225	0.78218874717790
H	2.19932282457408	-0.40592052662794	1.72050024060986
C	0.52944481710974	-0.91118730285313	-5.16885004782351
C	0.27460451158819	-0.94775042320192	-6.53777069192387
H	-0.39980028729643	-0.22927170114241	-6.98350735817895
C	0.90660200133308	-1.92622211291341	-7.27749203956322

H	0.73582502150897	-1.99232686537130	-8.34534998249822
C	1.75794954445711	-2.82430960140380	-6.64166931858252
H	2.26755811471733	-3.60479269647240	-7.19059198147187
C	1.93732219095542	-2.69453058347055	-5.27832584836304
H	2.58959344435217	-3.37136224607498	-4.73604397309404
C	-0.60176446267037	-1.46270865613919	-1.94660314255617
C	0.25018694519402	-2.50586807888706	-1.55846437605388
H	1.31916532827699	-2.41109384873024	-1.70022825785761
C	-0.26043025971325	-3.66740529969613	-1.00518544192377
H	0.41312432449927	-4.45893527498125	-0.70052331951564
C	-1.63008999537809	-3.81434236118000	-0.83652574845937
H	-2.02714457196248	-4.72118916912287	-0.39646893598517
C	-2.49147643799797	-2.79900201046989	-1.22835203802435
H	-3.56096067028283	-2.91251289177698	-1.10027875882007
C	-1.98368589183120	-1.63371644652489	-1.77705248646177
H	-2.67163000113451	-0.84999411364529	-2.07423259747234

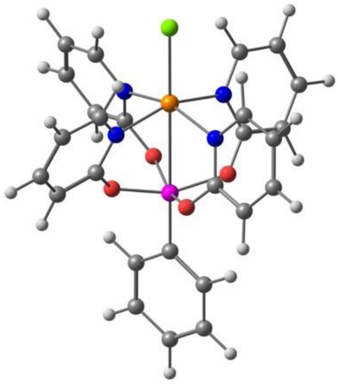

Figure S10. Optimized molecular structure of **3Si**.

PBE0:

final single point energy: -3939.123492373098 a.u.

final Gibbs free energy: -3938.74607607 a.u.

Table S6. Atomic coordinates for optimized structure of **3Si**.

Cu	-0.03167500586785	-0.02923288838029	0.00057004828175
Cl	-0.03977668083347	-0.06608922536165	2.40119721319554
Si	-0.02353221082056	0.01197628438636	-2.85163354601148
O	0.67765644280050	1.57301620017598	-2.46248046583884
O	1.54319029959260	-0.69089276892011	-2.49995279685700
O	-0.72732961206804	-1.55956715095892	-2.51101030387678
O	-1.59296893962430	0.70443315753314	-2.49207819900511
N	-0.03759323676301	1.97588716633671	-0.33154646062696
N	1.97116117781228	-0.01613311382514	-0.35978481675170
N	-0.02002755463602	-2.02465967929355	-0.39044855287626
N	-2.03123390572585	-0.03386724997376	-0.37493892559681
C	0.34042413681944	2.43010043160461	-1.53159498955160
C	0.41878473218076	3.80319139458208	-1.78652085559044
H	0.73411702781783	4.13109260228817	-2.76735758835895
C	0.08481102429097	4.68655367120799	-0.78686190019809
H	0.13742382691615	5.75327707916732	-0.96892413397431
C	-0.32614449378096	4.20194517714547	0.45350263573374
H	-0.59981573360501	4.86592592882771	1.26136025714048
C	-0.37132849379072	2.84045242657218	0.63513433795006
H	-0.66187232159902	2.38903487327713	1.57594168937557
C	2.41109803052417	-0.36741255924894	-1.57310082662695
C	3.78074465268398	-0.43287979524782	-1.84825145508139
H	4.09732932399649	-0.72713145273970	-2.83939228949103
C	4.67620478922399	-0.11544300909102	-0.85375417508141
H	5.74062866382906	-0.15913756949462	-1.05104708329910
C	4.20639705397106	0.26707588838329	0.40110157376365
H	4.87997311405415	0.52665100005843	1.20567281392661
C	2.84714664597010	0.30182898433295	0.60178013765920
H	2.40711227747861	0.57147169045141	1.55415209601515
C	-0.39064750758027	-2.44375221115478	-1.60548224204007
C	-0.46092382886982	-3.80873651411424	-1.90269422855944
H	-0.77108888693622	-4.10812904456593	-2.89423866370887
C	-0.12512831909368	-4.72062108881016	-0.92949478945902

H	-0.17088144137592	-5.78154121563705	-1.14446773350269
C	0.27929192893948	-4.27211154021930	0.32645459835331
H	0.55442515594281	-4.95915410376498	1.11429773854089
C	0.31556645836857	-2.91661524979012	0.55022012384469
H	0.60000264606732	-2.49266075537196	1.50551843754416
C	-2.46529136956546	0.35137168912249	-1.58000380917416
C	-3.83341196446378	0.41960696484399	-1.86174240116616
H	-4.14477488928091	0.74192608510350	-2.84577753320746
C	-4.73373925576311	0.06825928233707	-0.88315367889617
H	-5.79708035492963	0.11277771013859	-1.08598732218385
C	-4.27001166192253	-0.34960094573524	0.36266970646026
H	-4.94732733243933	-0.63660454617228	1.15468804396390
C	-2.91192502545242	-0.38423237955770	0.57102634493658
H	-2.47664865585134	-0.68066769959292	1.51757795176690
C	-0.01625938582289	0.03412646836880	-4.70716341807586
C	1.14863966689736	0.32763745023340	-5.41651423450900
H	2.06369309649254	0.54705554593370	-4.87768475372881
C	1.15814045044492	0.34199841340440	-6.80396687319115
H	2.07319712176337	0.57401947327621	-7.33654861979250
C	-0.00302222021719	0.05643787763711	-7.50880027211695
H	0.00225750710085	0.06460335620496	-8.59254263693571
C	-1.17087889583588	-0.23986519067985	-6.81960995120784
H	-2.08071920967708	-0.46391057422488	-7.36440551856735
C	-1.17444061833663	-0.24770377874372	-5.43208095592322
H	-2.09421612045041	-0.47692968326450	-4.90547370581184

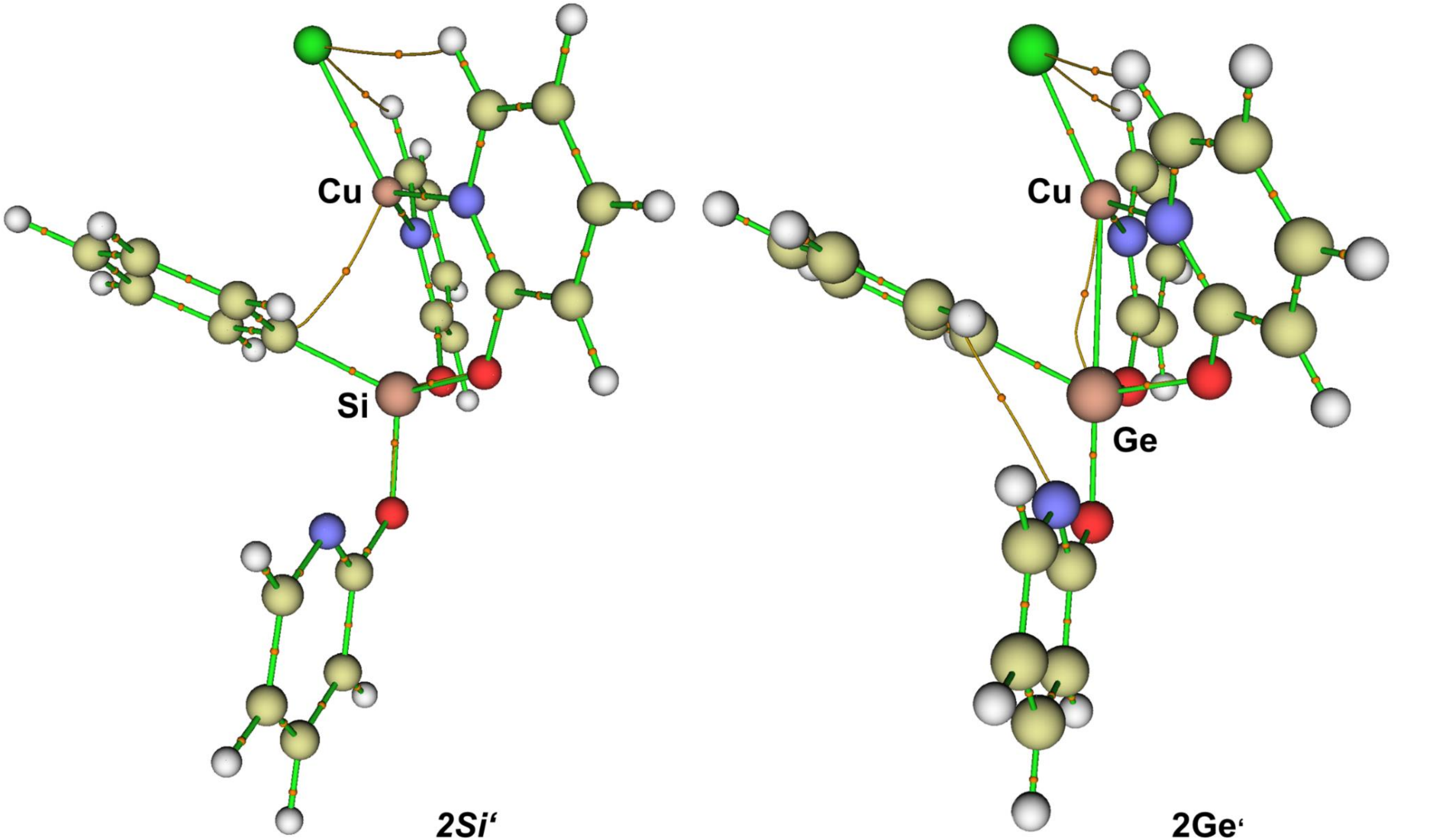

Figure S11. Bond paths with Bond Critical Points identified for the optimized molecular structures of ***2Si’*** and **2Ge’**.

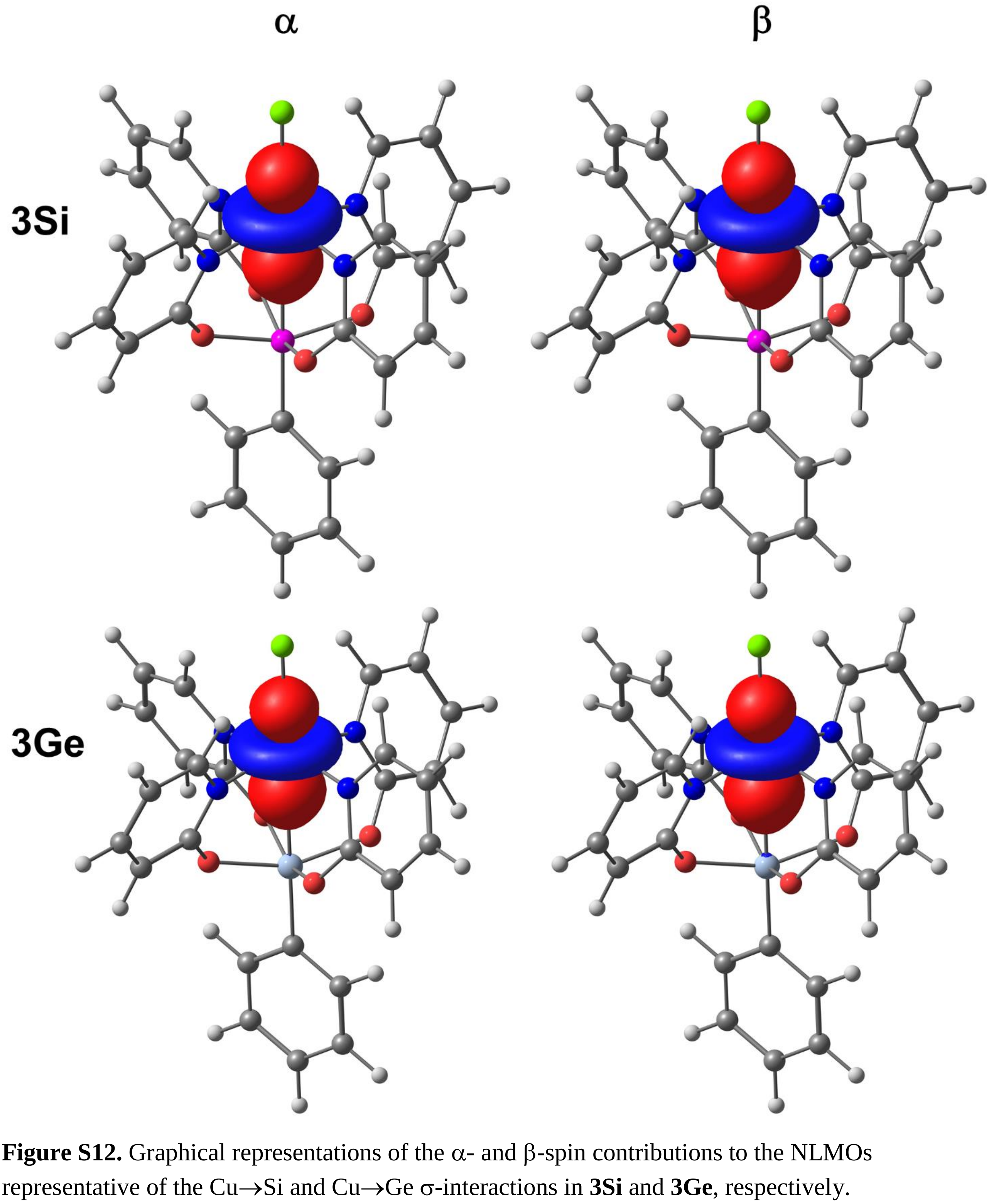

Figure S12. Graphical representations of the α- and β-spin contributions to the NLMOs representative of the Cu→Si and Cu→Ge σ-interactions in **3Si** and **3Ge**, respectively.

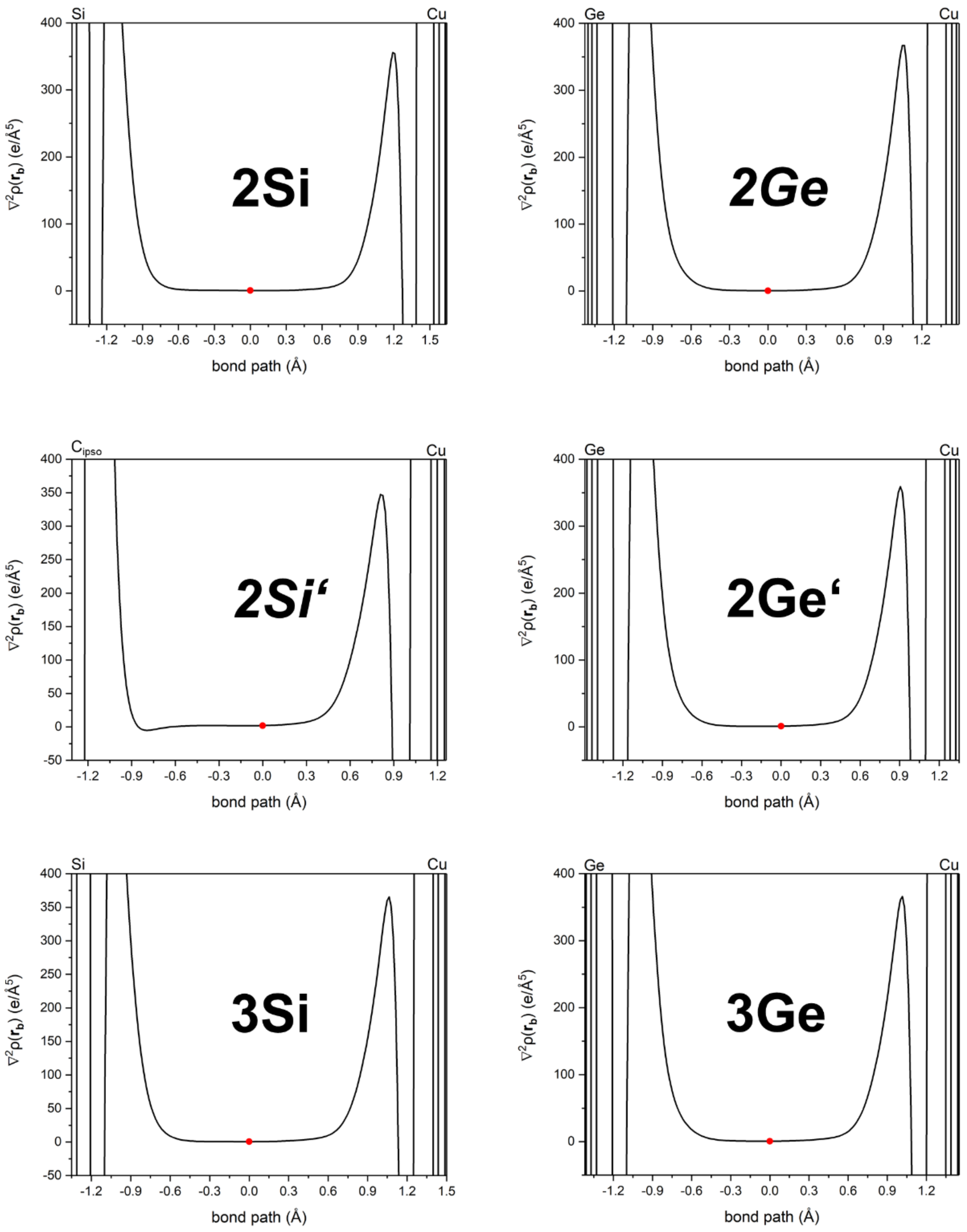

Figure S13. 1D plots of the Laplacian of electron density along the Cu–*E* (*E* = tetrel) bond path in ***2Ge***, **2Ge'**, **3Ge**, **2Si**, ***2Si'***, **3Si**. (The red dot at 0.0 indicates the position of the BCP. Note: In compound ***2Si'*** this bond path approaches C_{ipso} rather than Si, as shown in Figure S11)
